# Far upstream element‐binding protein 1 confers lobaplatin resistance by transcriptionally activating PTGES and facilitating the arachidonic acid metabolic pathway in osteosarcoma

**DOI:** 10.1002/mco2.257

**Published:** 2023-05-09

**Authors:** Qiong Ma, Jin Sun, Huan Wang, Chengpei Zhou, Chenyu Li, Yonghong Wu, Yanhua Wen, Xiaoyu Zhang, Xingguang Ren, Zheng Guo, Li Gong, Wei Zhang

**Affiliations:** ^1^ Department of Pathology Tangdu Hospital Air Force Medical University Xi'an China; ^2^ Orthopedic Oncology Institute Department of Orthopedic Surgery Tangdu Hospital Air Force Medical University Xi'an China

**Keywords:** arachidonic acid metabolic pathway, FUBP1, lobaplatin resistance, osteosarcoma, PTGES

## Abstract

Drug resistance is a major obstacle in cancer treatment and recurrence prevention and leads to poor outcomes in patients suffering from osteosarcoma. Clarification of the mechanism of drug resistance and exploration of effective strategies to overcome this obstacle could lead to clinical benefits for these patients. The expression of far upstream element‐binding protein 1 (FUBP1) was found to be markedly elevated in osteosarcoma cell lines and clinical specimens compared with osteoblast cells and normal bone specimens. High expression of FUBP1 was correlated with a more aggressive phenotype and a poor prognosis in osteosarcoma patients. We found that overexpression of FUBP1 confers lobaplatin resistance, whereas the inhibition of FUBP1 sensitizes osteosarcoma cells to lobaplatin‐induced cytotoxicity both in vivo and in vitro. Chromatin immunoprecipitation‐seq and RNA‐seq were performed to explore the potential mechanism. It was revealed that FUBP1 could regulate the transcription of prostaglandin E synthase (PTGES) and subsequently activate the arachidonic acid (AA) metabolic pathway, which leads to resistance to lobaplatin. Our investigation provides evidence that FUBP1 is a potential therapeutic target for osteosarcoma patients. Targeting FUBP1, its downstream target PTGES and the AA metabolic pathway may be promising strategies for sensitizing chemoresistant osteosarcoma cells to lobaplatin.

## Graphical abstract

High expression of far upstream element‐binding protein 1 (FUBP1) on tumor cells promotes lobaplatin chemoresistance in osteosarcoma cells. FUBP1 binds to the promoter of PTGES and accordingly regulates the transcription process, which activates arachidonic acid signaling and leads to lobaplatin chemoresistance.

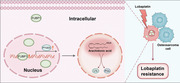

## 1 INTRODUCTION

Osteosarcoma is the most common primary malignant bone tumor, which mainly develops in the long bones and has a poor prognosis.^1^ The current treatment for osteosarcoma is a combination of aggressive surgical resection and chemotherapy.^2^ Platinum‐based chemotherapeutics, including cisplatin, oxaliplatin, and lobaplatin, are commonly used in the clinic against osteosarcoma.^3^ Lobaplatin is a third‐generation platinum‐based antitumor agent that shows fewer side effects and better antitumor activity.^4^ However, recurrence and metastasis remain difficult problems for patients after several courses of treatment. Most patients who succumb to osteosarcoma exhibit marked chemoresistance. This has motivated efforts to acquire a better understanding of the molecular mechanism of chemoresistance in osteosarcoma, which would potentially provide novel opportunities for patients.

Arachidonic acid (AA) is a 20‐carbon fatty acid with 4 double bonds.^5^ Evidence has demonstrated that this tetra‐unsaturated fatty acid plays a pivotal role in not only normal cellular membrane fluidity but also numerous enzymatic processes that yield active lipid mediators.^6^ In addition, the polyunsaturated fatty acid biosynthesis pathway, which includes AA, plays an essential role in ferroptosis in gastric cancer.^7^ Regarding resistance to chemotherapeutics, Cioce et al. reported increased levels of AA in transformed mesothelioma cells treated with pemetrexed and concluded that AA is an early mediator in the adaptation to pemetrexed in malignant pleural mesothelioma.^8^ More importantly, suppressing the AA pathway could hinder the chemotherapy‐induced repopulation of ovarian cancer cells, which indicates that inhibition of the AA pathway might have therapeutic potential.^9^ However, no investigation has shown the role of AA metabolism in lobaplatin resistance in human osteosarcoma, and the molecules that can regulate the activation of the AA pathway in the above process are still unknown.

As a multifunctional DNA‐ and RNA‐binding protein, far upstream element‐binding protein 1 (FUBP1) mainly functions in transcription processes and regulates the expression of its target genes. There is increasing evidence of the oncogenic role of FUBP1 in several types of tumors, including hepatocellular carcinoma,^10^ gastric cancer,^11^ neuroblastoma,^12^ leukemia,^13^ and nasopharyngeal carcinoma.^14^ The molecular basis and signal transduction pathway by which FUBP1 contributes to the progression of tumors are currently being investigated. The well‐known oncogene MYC was determined to be a downstream gene regulated by FUBP1 in several independent investigations. It was reported that FUBP1 can activate MYC transcription to promote proliferation, invasion, and metastasis in some types of human cancer cells^15^ and enforce the epigenetic setpoint for MYC expression in primary single murine cells.^16^ Regarding chemotherapy resistance, knockdown of FUBP1 was found to decrease the migration and metastasis of triple‐negative breast cancer cells and enhance their sensitivity to cisplatin.^17^ On the other hand, FUBP1 knockdown was reported to enhance bromodomain and extraterminal domain inhibitor resistance in pancreatic cancer cells by decreasing the expression of c‐Myc.^18^ These investigations highlight that FUBP1 could be used as a therapeutic candidate for patient‐tailored treatment alongside chemotherapy for different tumors. Our previous research found that FUBP1 could decrease the sensitivity of osteosarcoma cells to lobaplatin.^19^ However, the specific mechanisms and signaling pathway remain unclear.

Prostaglandin E synthase (PTGES) is a critical synthase involved in prostaglandin E2 (PGE2) biosynthesis in AA metabolism. It is usually expressed at low levels when cells are not under stress. However, inflammation and the presence of cancer cells can rapidly induce high PTGES expression.^20^ PTGES is considered a valuable target in the treatment of inflammation and cancers.^21^ However, the role of PTGES in chemoresistance is unclear.

In this study, we explored the effects of FUBP1 in lobaplatin resistance both in vitro and in vivo. Chromatin immunoprecipitation (ChIP)‐seq and RNA‐seq were performed to explore the potential mechanism. This led us to identify the PTGES and the AA metabolic pathway as probable targets of reduced susceptibility to lobaplatin and the markers to predict lobaplatin responsiveness and clinical outcomes. Luciferase assay and rescue experiments verified that FUBP1 could transcriptionally bind to the promoter of PTGES and promote lobaplatin resistance. Truncation assay revealed the binding fragments. In addition, both the selective PTGES inhibitor CAY10526 and EP2 agonist Evatanepag were used to confirm the contribution of FUBP1 to lobaplatin resistance by means of AA metabolism. Our study indicated the potential therapeutic value of FUBP1 and AA metabolism for increasing the sensitivity to lobaplatin in patients with osteosarcoma.

## 2 RESULTS

### 2.1 FUBP1 overexpression correlates with human osteosarcoma progression and drug resistance

**FIGURE 1:**
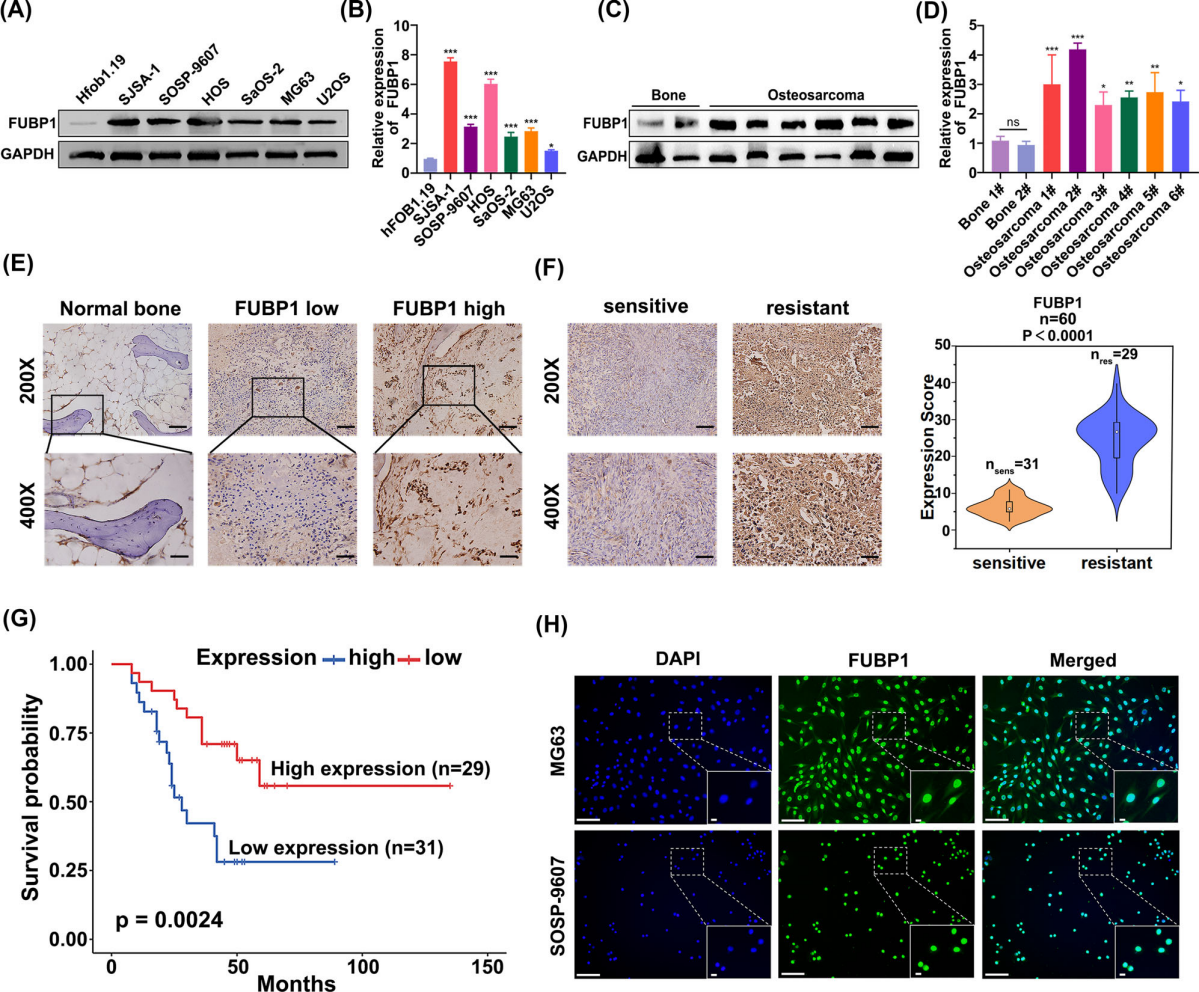
High far upstream element‐binding protein 1 (FUBP1) expression was correlated with human osteosarcoma progression and a poor prognosis. (A) Western blotting analysis of FUBP1 expression in the osteoblast cell line human fetal osteoblasts (hFOB) 1.19 and six cultured osteosarcoma cell lines. GAPDH was used as a protein loading control. (B) The expression of FUBP1 was detected in the osteoblast cell line hFOB 1.19 and six cultured osteosarcoma cell lines by RT‐PCR. *p < 0.05, **p < 0.01, ***p < 0.001. (C) Western blotting analysis of FUBP1 expression in six primary osteosarcoma tissues and their matched adjacent normal bone tissues. GAPDH was used as a protein loading control. (D) Expression of FUBP1 was detected in osteosarcoma tissues and their matched adjacent normal bone tissues by RT‐PCR. (E) Immunohistochemistry (IHC) staining indicating the FUBP1 protein levels in human osteosarcoma tissues compared with normal bones. Scale bars = 100 µm (×200), scale bars = 50 µm (×400). (F) The expression of FUBP1 in platinum‐sensitive and platinum‐resistant osteosarcoma tissues using IHC staining. Scale bars = 100 µm (×200), scale bars = 50 µm (×400). (G) Kaplan‒Meier overall survival curves comparing osteosarcoma patients with low and high FUBP1 expression levels (*n* = 60; *p* < 0.01). (H) Immunofluorescence staining showed that FUBP1 was predominantly located in the nuclei of MG63 and SOSP‐9607 osteosarcoma cells (scale bars = 200 µm, scale bars = 12.5 µm).

We compared the expression of FUBP1 in osteosarcoma cell lines, an osteoblast cell line, osteosarcoma tumor tissues, and nontumorous bone tissues at both the protein and mRNA levels. FUBP1 was clearly upregulated in the six different osteosarcoma cell lines and six osteosarcoma tumor tissues compared to the osteoblast cell line human fetal osteoblasts (hFOB) 1.19 and normal bone tissues (Figure 1A–D). To further investigate the clinical significance of FUBP1 in osteosarcoma patients, a cohort of 60 patients who were treated with platinum‐based chemotherapy was included (Table S1). Immunohistochemistry (IHC) analysis showed that FUBP1 was markedly upregulated in osteosarcoma tissues compared with normal bone tissues (Figure 1E). In addition, specimens resistant to platinum‐based chemotherapy demonstrated stronger positive expression of FUBP1 than the sensitive specimens (*n* = 60, *n*_sens_ = 31, *n*_res_ = 29, Figure 1F). The survival analysis demonstrated that high expression of FUBP1 correlated with poor overall patient survival (Figure 1G). Furthermore, FUBP1 was mainly localized in the nuclei of osteosarcoma cells according to the results of the immunofluorescence (IF) assay (Figure 1H) and the fluorescence in situ hybridization (FISH) assay (Figure S1). These results suggest that FUBP1 has potential clinical value for predicting disease outcome in osteosarcoma.

### 2.2 FUBP1 confers lobaplatin resistance in osteosarcoma in vitro and in vivo

**FIGURE 2:**
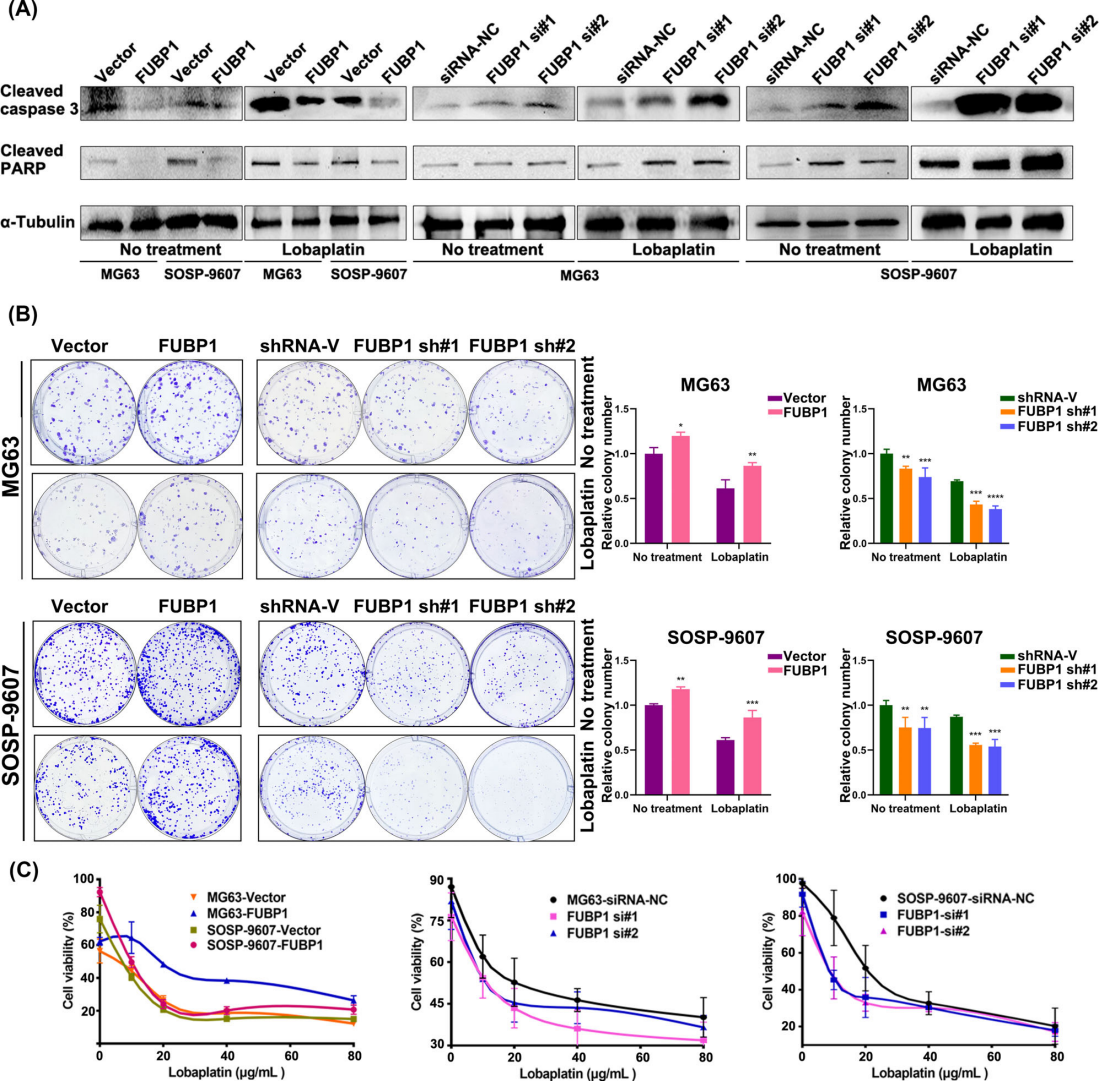
Far upstream element‐binding protein 1 (FUBP1) conferred lobaplatin resistance in osteosarcoma in vitro. (A) Western blotting analysis of cleaved caspase 3 and cleaved poly (ADP‐ribose) polymerase (PARP) in the indicated cells; α‐tubulin was used as a loading control. (B) Colony formation assays were used to determine the proliferation ability of the indicated cells. Representative images and quantification are shown. (C) CCK‐8 assays were performed to determine the viabilities of the indicated cells. Cells without any treatment were considered the control. *p < 0.05, **p < 0.01, ***p < 0.001, ****p < 0.0001. shRNA‐V, scrambled shRNA.

Drug‐resistant tumor cells show abnormal regulation of proliferation and apoptosis. To investigate the role of FUBP1 in osteosarcoma cells, osteosarcoma cell lines in which FUBP1 was overexpressed or knocked down were constructed (Figure S2). The protein levels of cleaved caspase 3 and cleaved poly (ADP‐ribose) polymerase (PARP) were significantly decreased in FUBP1‐overexpressing osteosarcoma cells. In the FUBP1 knockdown cells transfected with the small interfering RNA (siRNA), the trends were the opposite (Figure 2A, Figure S3). The proportion of apoptotic cells in FUBP1‐overexpressing osteosarcoma cells treated with lobaplatin was markedly reduced, whereas this proportion was increased in FUBP1‐silenced osteosarcoma cells (Figure S4A,B). The colony formation assay showed that the colony formation efficiency of FUBP1‐overexpressing osteosarcoma cells (MG63 and SOSP‐9607) treated with lobaplatin increased, whereas that of FUBP1‐silenced cells declined sharply compared with that of the control cells (Figure 2B, *p* < 0.05). Moreover, the IC_50_ values for lobaplatin were 14.95 µg/mL (MG63‐Vector) and 28.36 µg/mL (MG63‐FUBP1) for the FUBP1‐overexpressing MG63 cells and 7.94 µg/mL (SOSP‐9607‐Vector) and 10.04 µg/mL (SOSP‐9607‐FUBP1) for FUBP1‐overexpressing SOSP‐9607 cells. For the FUBP1 knockdown tumor cells, the IC_50_ values were 10.69 µg/mL (MG63‐siRNA‐NC), 7.25 µg/mL (FUBP1 si#1), and 8.05 µg/mL (FUBP1 si#2) for the MG63 cells and 18.26 µg/mL (SOSP‐9607‐siRNA‐NC), 10.45 µg/mL (FUBP1 si#1), and 10.33 µg/mL (FUBP1 si#2) for the SOSP‐9607 cells (Figure 2C). The significant increases in IC_50_ values in FUBP1‐overexpressing osteosarcoma cells and decreases in IC_50_ values in FUBP1‐silenced cells versus control cells suggest that FUBP1 enhances the resistance of osteosarcoma cells to lobaplatin in vitro. These results revealed that dysregulation of FUBP1 is involved in lobaplatin resistance in osteosarcoma cells.

**FIGURE 3:**
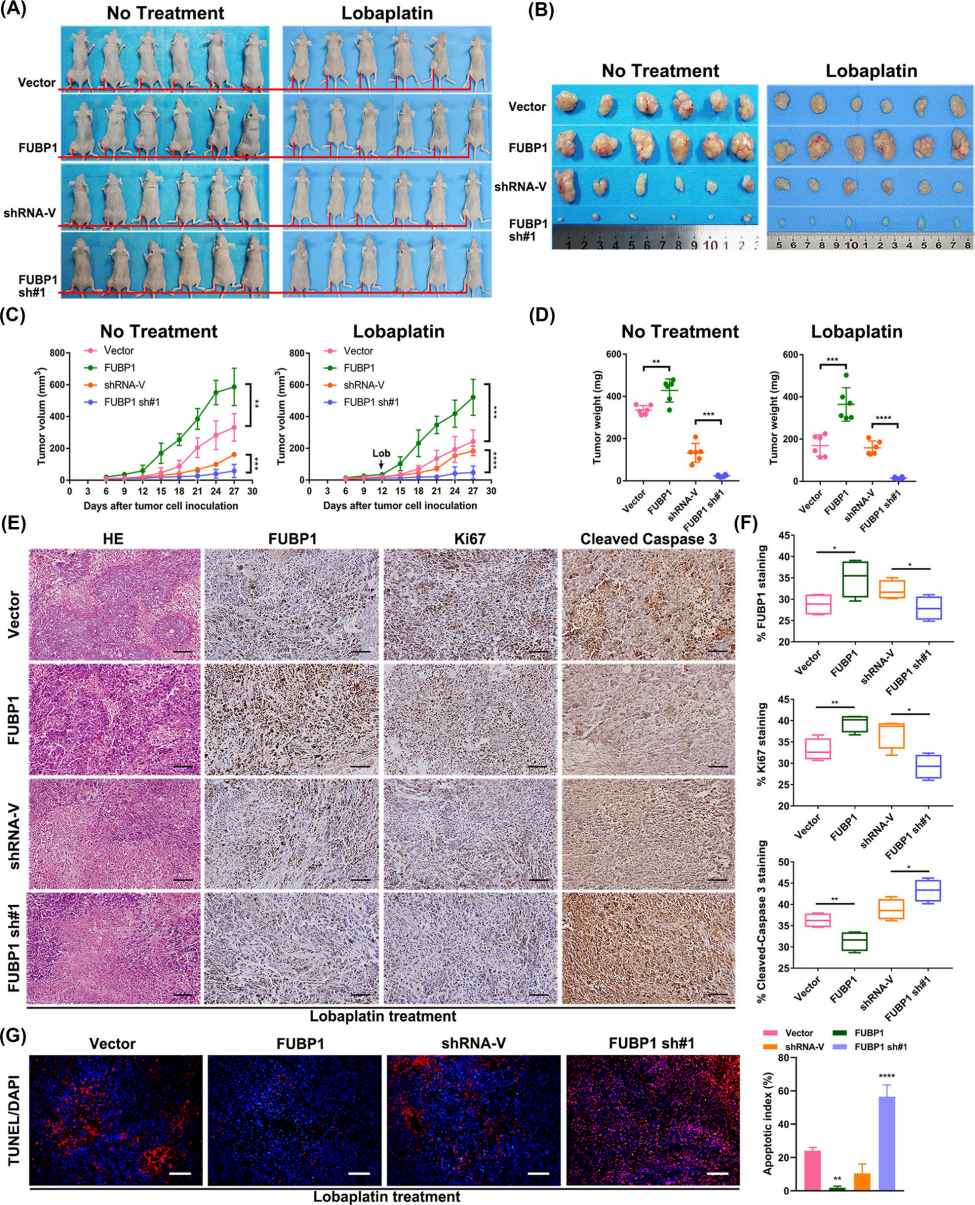
Far upstream element‐binding protein 1 (FUBP1) conferred lobaplatin resistance in osteosarcoma in vivo. (A) Representative images of tumor‐bearing mice inoculated with the indicated cells and then treated with or without lobaplatin. (B) All tumors were acquired from nude mice in each group. (C) Tumor volumes were measured and recorded on the indicated days. (D) Tumor weights were recorded and are indicated as the means ± standard error of the means (SEMs). (E) Hematoxylin–eosin staining and immunohistochemistry staining with antibodies against FUBP1, Ki67, and cleaved caspase‐3 were performed on xenograft tumor tissue sections from the lobaplatin‐treatment group (scale bars = 100 µm). (F) Box‐and‐whisker plots showing the mean ± SEM values for Ki67 and cleaved caspase 3 staining from each group of lobaplatin‐treated mice (*n* = 6). (G) After fixation with paraformaldehyde, TdT‐mediated dUTP nick end labeling (TUNEL) staining assays demonstrated cell apoptosis in the indicated tissues (scale bars = 100 µm). *p < 0.05, **p < 0.01, ***p < 0.001, ****p < 0.0001.

To validate the function of FUBP1 in vivo, a nude mouse xenograft model was utilized to assess the effect of FUBP1 on osteosarcoma chemoresistance. Nude mice were inoculated subcutaneously with control overexpression vector, FUBP1‐overexpression lentivirus, vector control‐short hairpin RNA (shRNA), or FUBP1 shRNA using both MG63 and SOSP‐9607 cells. Lobaplatin treatment was initiated once every 3 days at a dosage of 3 mg/kg once the tumor volume reached 0.125 cm^3^. At the end of treatment, the mice were sacrificed, and the tumors were harvested and examined (Figure 3A,B). The MG63 tumor volumes in the FUBP1‐overexpressing mice were significantly larger than those in the vector‐overexpressing mice after lobaplatin treatment. A significant reduction in tumor weights was observed in FUBP1 knockdown xenografts upon lobaplatin treatment compared with the tumors of control mice inoculated with vector control shRNA (Figure 3C,D). FUBP1 played a similar role in SOSP‐9607 osteosarcoma cells (Figure S5). We also found that the differences in tumor weights and volumes were remarkably larger in the lobaplatin‐treatment group than in the group without lobaplatin treatment. These results suggest that FUBP1 confers osteosarcoma resistance to lobaplatin in vivo.

Quantification of cell proliferation and apoptosis in MG63 and SOSP‐9607 xenografts was performed using IHC staining. The results showed that upon lobaplatin treatment for 4 weeks, FUBP1‐overexpressing xenografts derived from two different osteosarcoma cell lines displayed higher expression of FUBP1 and Ki67 than the vector‐overexpressing mice and lower expression of cleaved caspase 3 than the control xenografts, whereas FUBP1 knockdown xenografts derived from two different osteosarcoma cell lines demonstrated weak FUBP1 and Ki67 staining and more cleaved caspase 3 activation (Figure 3E,F, Figure S6A,B). These results indicate that apoptosis was induced by lobaplatin in each group, and FUBP1‐overexpressing xenografts showed more proliferative and fewer apoptotic tumor cells than the vector‐overexpressing and FUBP1‐silenced xenografts, which resulted in resistance to lobaplatin (Figure 3G, Figure S6C).

### 2.3 ChIP‐seq and RNA‐seq reveal the probable downstream genes of FUBP1

**FIGURE 4:**
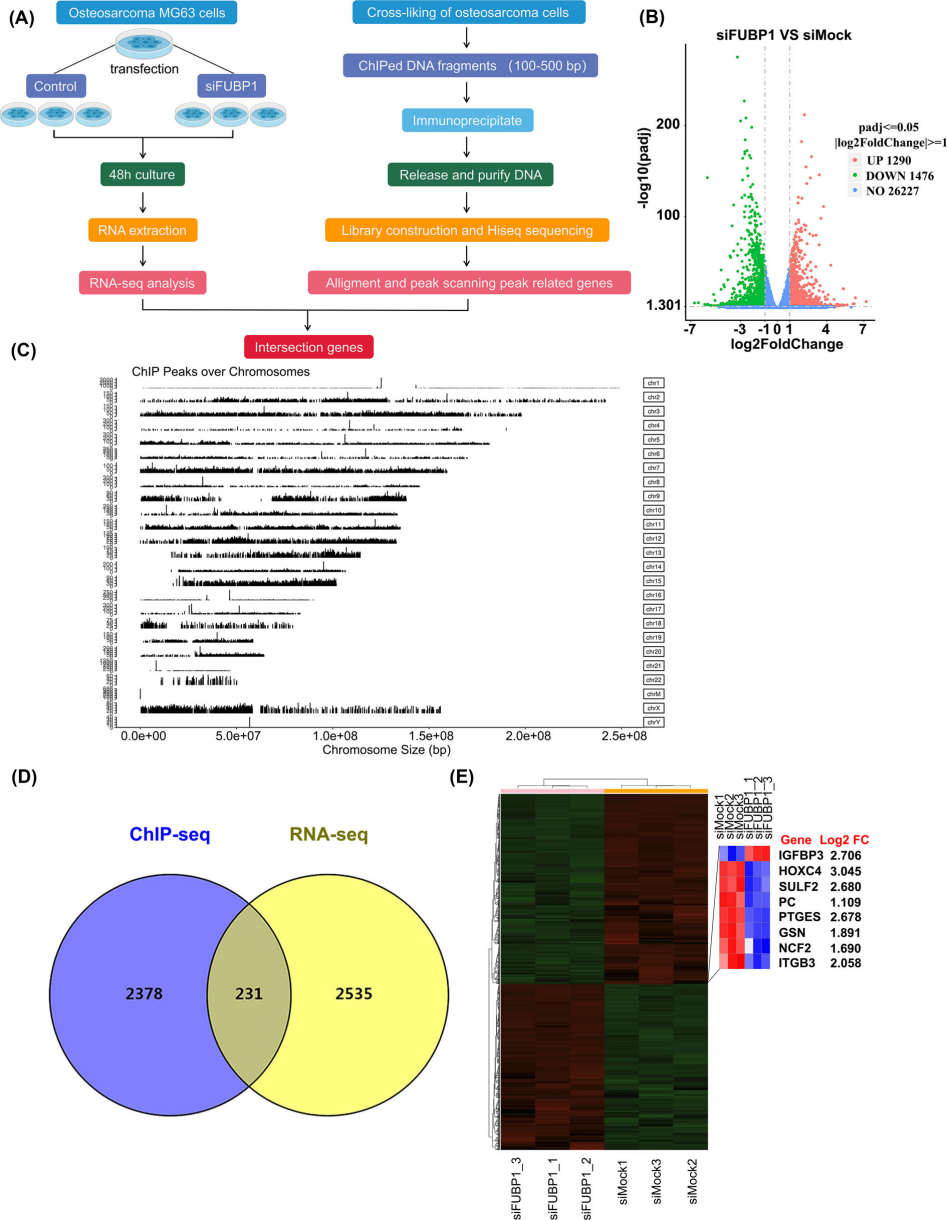
The key downstream genes of far upstream element‐binding protein 1 (FUBP1) were enriched using chromatin immunoprecipitation (ChIP)‐seq and RNA‐seq. (A) ChIP‐seq enrichment and RNA‐seq analysis workflow. (B) RNA‐seq analysis of the differentially expressed mRNAs between FUBP1‐knockdown and Ctrl‐treated MG63 osteosarcoma cells. The red dots represent upregulation, the green dots represent downregulation, and the blue dots represent genes whose fold change was less than twofold. A total of 28,993 genes were identified, including 1290 upregulated genes and 1476 downregulated genes (|log2 fold change| > 1, *p* < 0.05). (C) ChIP‐seq demonstrated the ChIP peaks over chromosomes using the FUBP1 antibody. (D) Venn diagram comparing the differentially expressed genes identified by RNA‐seq and those identified by ChIP‐seq. (E) A heatmap showing the mRNA expression levels of candidate genes in FUBP1 knockdown or Ctrl‐treated MG63 osteosarcoma cells.

**FIGURE 5:**
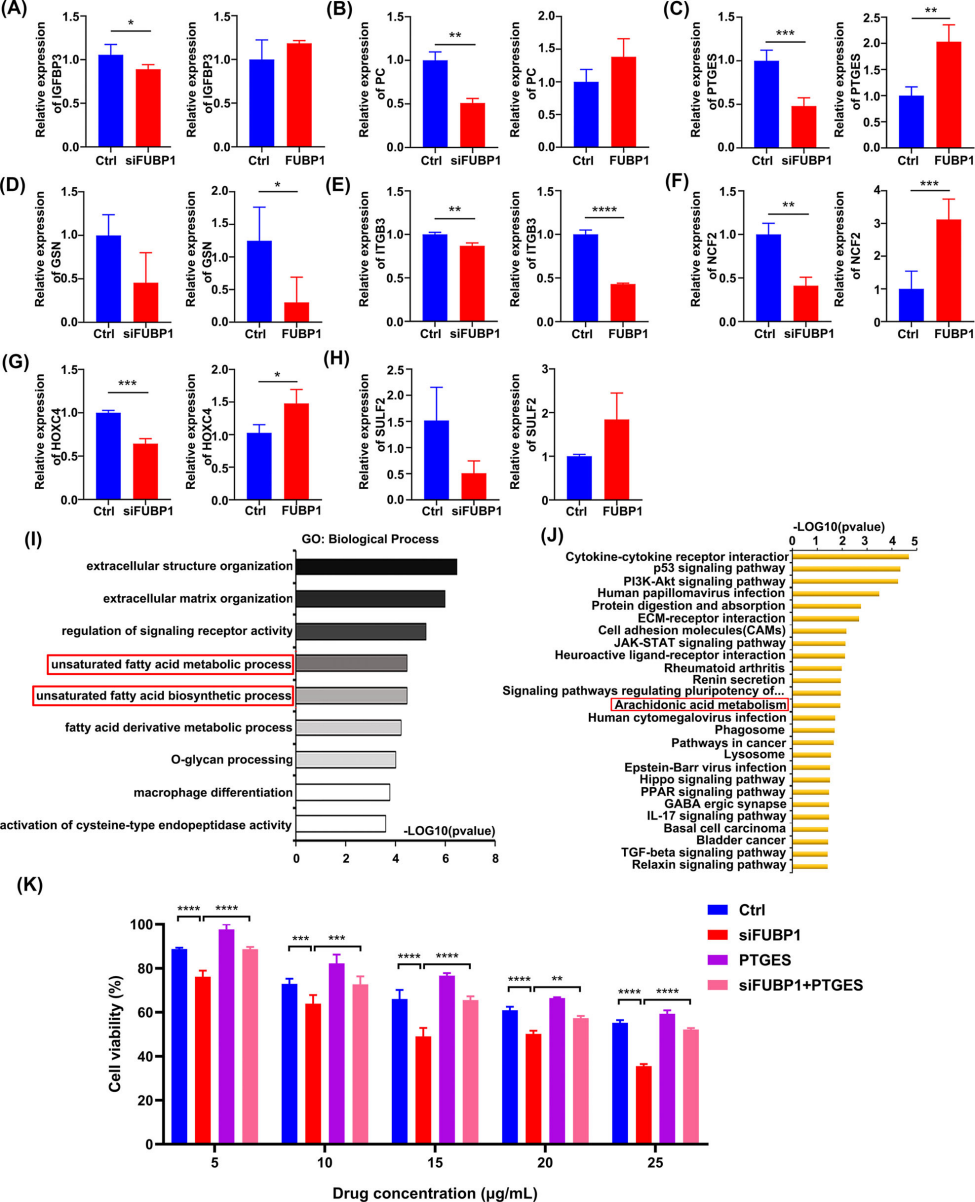
Real‐time PCR and informatics analysis were conducted to detect the candidate downstream genes of far upstream element‐binding protein 1 (FUBP1). (A) IGFBP3; (B) PC; (C) PTGES; (D) GSN; (E) ITGB3; (F) NCF2; (G) HOXC4; and (H) SULF2. GAPDH was used as a control. (I) Gene ontology analysis was performed to identify the enriched biological processes in FUBP1 knockdown osteosarcoma cells. The unsaturated fatty acid metabolism process and the unsaturated fatty acid biosynthesis process were both enriched. (J) Kyoto Encyclopedia of Genes and Genomes (KEGG) analysis was conducted to identify enriched signaling pathways in the indicated cells. The arachidonic acid metabolism pathway was significantly enriched. (K) Osteosarcoma cells transfected with siFUBP1 and Ptges‐overexpression plasmids showed a significant elevation in chemoresistance to lobaplatin compared with that in cells transfected with only siFUBP1. *p < 0.05, **p < 0.01, ***p < 0.001, ****p < 0.0001.

To identify the key downstream target of FUBP1, ChIP‐seq and RNA‐seq were performed using the human osteosarcoma cell line MG63 (Figure 4A–D, Figure S7). Genes near the transcriptional start site (TSS), specifically from 3000 bp upstream to 500 bp downstream, were acquired, and genes that overlapped with the differentially expressed RNA‐seq genes (|log_2_ fold change| > 1, *p* value <0.05) were selected, narrowing the pool to 231 genes (Figure 4E). Real‐time PCR was performed to verify the expression of genes potentially downstream of FUBP1, which included IGFBP3, PC, PTGES, GSN, ITGB3, NCF2, HOXC4, and SULF2 (Table S2). The expression trends of PTGES, NCF2, and HOXC4 after FUBP1 overexpression by plasmid transfection or FUBP1 downregulation by siRNA transfection were consistent with those in the RNA‐seq results (Figure 5A–H).

According to the bioinformatics analysis, we found that the enriched gene ontology (GO) terms included unsaturated fatty acid metabolic process (GO: 0033559, *p* = 3.38E − 05) and unsaturated fatty acid biosynthetic process (GO: 0006636, *p* = 3.43E − 05) in the category of biological process (Figure 5I, Table S3). Interestingly, the Kyoto Encyclopedia of Genes and Genomes (KEGG) pathway analysis demonstrated that AA metabolism was significantly enriched (hsa00590, *p* = 0.012) (Figure 5J, Table S4). All the above GO and KEGG terms suggest that PTGES may play a crucial role in the process of FUBP1‐induced chemoresistance.

As PTGES was the probable downstream gene of FUBP1, a PTGES‐overexpression plasmid was synthesized. Tumor cells were then cotransfected with small interfering oligonucleotides targeting FUBP1 and PTGES‐overexpression plasmids, and these tumor cells demonstrated increased cell viability compared with cells transfected with only FUBP1 knockdown nucleotides. The average viability of osteosarcoma cells transfected with both FUBP1 siRNA and the PTGES‐overexpression plasmid was markedly higher than that of cells transfected with only FUBP1 siRNA. Interestingly, the increases in viability were more significant when the cells were treated with different concentrations of lobaplatin (Figure 5K). These results indicate that FUBP1 may regulate osteosarcoma chemotherapy resistance through PTGES.

### 2.4 PTGES knockdown confers chemotherapy sensitivity in osteosarcoma

**FIGURE 6:**
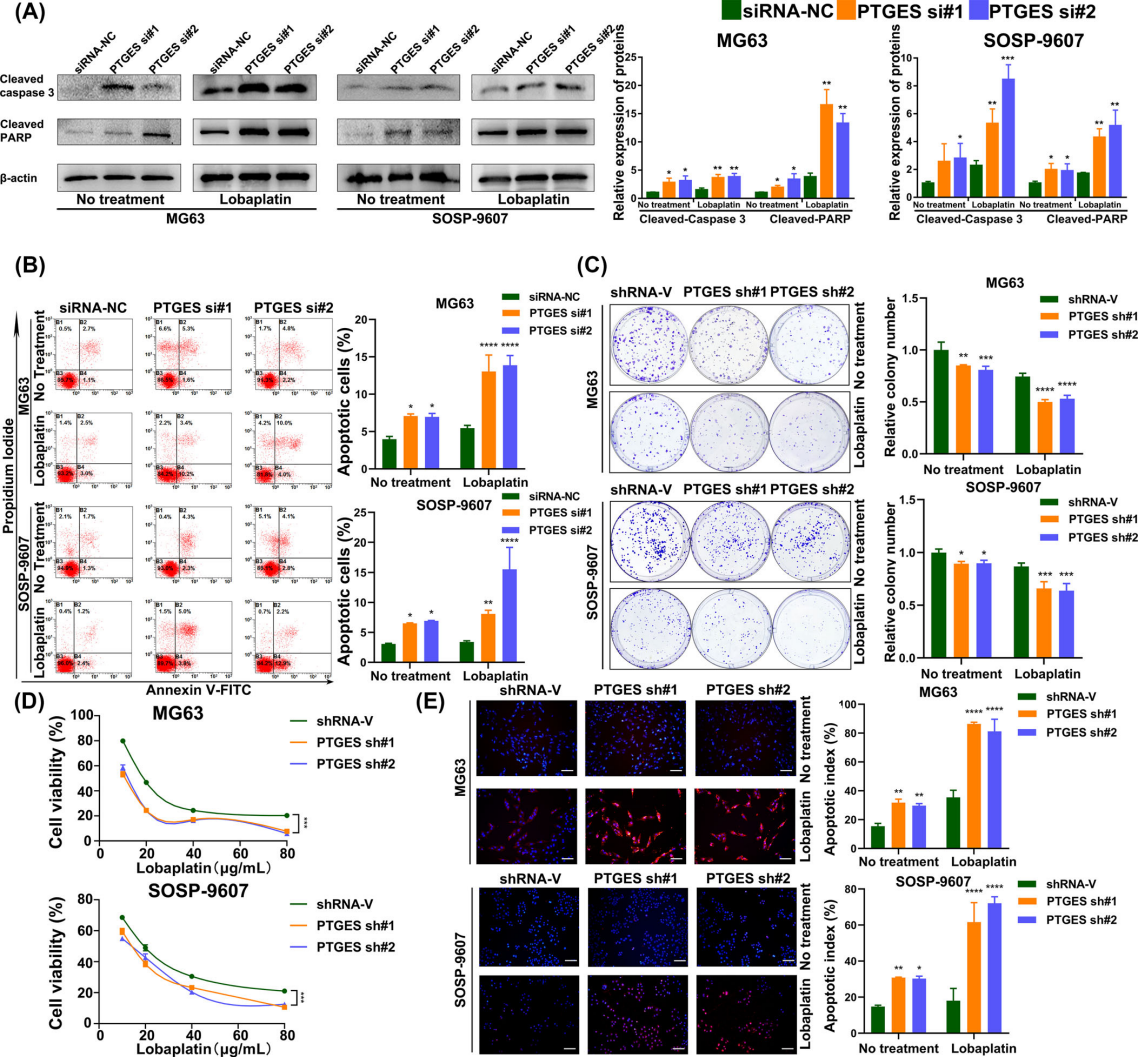
Knockdown of prostaglandin E synthase (PTGES) increased the sensitivity of osteosarcoma cells to lobaplatin in vitro. (A) Western blotting analysis of cleaved caspase 3 and cleaved poly (ADP‐ribose) polymerase (PARP) in osteosarcoma cells transfected with siPTGES; β‐actin was used as a housekeeping gene. (B) The apoptosis rates of osteosarcoma cells transfected with siPTGES and then treated with or without lobaplatin for 24 h were detected using Annexin V‐FITC and PI staining. (C) Colony formation assays were used to determine the proliferation ability of osteosarcoma cells transfected with shPTGES. (D) CCK‐8 assays were performed to determine the viability of osteosarcoma cells transfected with siPTGES and treated with different concentrations of lobaplatin. (E) TdT‐mediated dUTP nick end labeling (TUNEL) staining assays demonstrated the apoptosis of the indicated cells (scale bars = 100 µm). *p < 0.05, **p < 0.01, ***p < 0.001, ****p < 0.0001.

To determine the biological effects of PTGES knockdown in osteosarcoma cells, cells were transfected with the indicated siRNAs and shRNAs (Table S5, Figure S8). The protein levels of cleaved caspase 3 and cleaved PARP were elevated in PTGES‐knockdown osteosarcoma cells, and cells treated with lobaplatin demonstrated more significant differences in these proteins than those without lobaplatin treatment (Figure 6A). The proportion of apoptotic cells among the PTGES‐knockdown osteosarcoma cells treated with lobaplatin was markedly increased compared with that in the untreated group (Figure 6B). The colony formation assay demonstrated that the cloning efficiency of PTGES‐knockdown osteosarcoma cells declined more sharply in the lobaplatin group than in the untreated group (Figure 6C). In addition, the IC_50_ values for lobaplatin were 15.68 µg/mL (shRNA‐V), 14.59 µg/mL (PTGES sh#1), and 13.72 µg/mL (PTGES sh#2) for MG63 cells and 15.56 µg/mL (shRNA‐V), 14.89 µg/mL (PTGES sh#1), and 14.99 µg/mL (PTGES sh#2) for SOSP‐9607 cells (Figure 6D), which indicates that PTGES knockdown enhances the sensitivity of osteosarcoma cells to lobaplatin in vitro. TdT‐mediated dUTP nick end labeling (TUNEL) staining of the indicated cells demonstrated a higher rate of apoptosis in PTGES‐knockdown osteosarcoma cells treated with lobaplatin than in the untreated group (Figure 6E). Taken together, these results indicate that knockdown of PTGES could sensitize osteosarcoma cells to lobaplatin.

### 2.5 AA metabolism is essential for FUBP1‐induced chemoresistance

**FIGURE 7:**
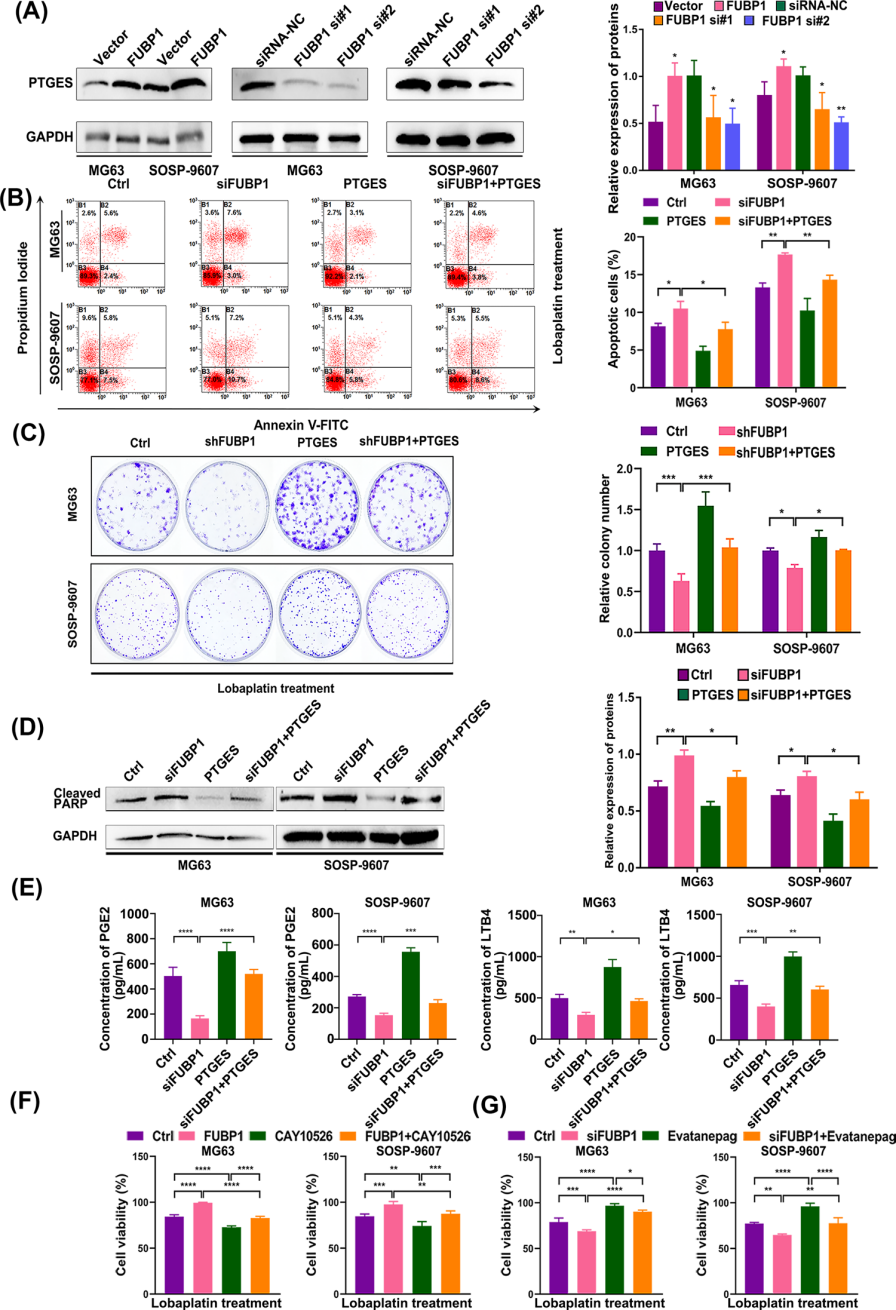
Far upstream element‐binding protein 1 (FUBP1) confers chemoresistance to osteosarcoma cells through the arachidonic acid metabolic pathway. (A) Western blotting analysis of prostaglandin E synthase (PTGES) in osteosarcoma cells transfected with FUBP1‐overexpressing plasmids or siFUBP1. (B) The effect of PTGES overexpression on the sensitivity of FUBP1 knockdown osteosarcoma cells treated with lobaplatin for 24 h detected using flow cytometry. (C) The effect of PTGES overexpression on the sensitivity of FUBP1 knockdown osteosarcoma cells treated with lobaplatin evaluated with a colony formation assay. (D) The effect of PTGES overexpression on the sensitivity of FUBP1 knockdown osteosarcoma cells treated with lobaplatin evaluated by western blotting using a cleaved poly (ADP‐ribose) polymerase (PARP) antibody. (E) The effect of PTGES overexpression on prostaglandin E2 (PGE2) and leukotriene B4 (LTB4) secretion in FUBP1 knockdown osteosarcoma cells evaluated by ELISA. (F) The effect of a selective PTGES inhibitor CAY10526 on the sensitivity of FUBP1‐overexpressing osteosarcoma cells treated with lobaplatin evaluated by CCK‐8 assays. (G) The effect of an EP2 receptor agonist Evatanepag on the sensitivity of FUBP1 knockdown osteosarcoma cells treated with lobaplatin evaluated by CCK‐8 assays. *p < 0.05, **p < 0.01, ***p < 0.001, ****p < 0.0001.

The necessity of PTGES activation to allow FUBP1 to enhance resistance to chemotherapy in osteosarcoma cells was further investigated. PTGES was dramatically elevated in FUBP1‐overexpressing cells but downregulated in FUBP1‐silenced osteosarcoma cells (Figure 7A). Silencing FUBP1 and upregulating PTGES at the same time could rescue the resistance ability of FUBP1 as determined by flow cytometry and colony formation assays. The average apoptosis rates were 8.2% ± 0.38%, 10.5% ± 0.95%, 4.9% ± 0.61%, and 7.8% ± 0.93% in the control cells, FUBP1‐interfering cells, PTGES‐overexpressing cells, and cotransfected cells, respectively (Figure 7B). Moreover, the cotransfected cells demonstrated increased colony formation compared with the FUBP1‐silenced cells (Figure 7C). Western blotting revealed that the expression level of cleaved PARP decreased in the cotransfected osteosarcoma cells compared with the FUBP1‐silenced cells when 20 µg/mL lobaplatin was administered to the tumor cells for 24 h (Figure 7D). Taken together, these results indicated that PTGES is the crucial downstream gene that is regulated by FUBP1 and contributes to chemoresistance.

To further validate that FUBP1 mediates the chemoresistance of osteosarcoma cells through AA metabolic pathway, PGE2 and leukotriene B4 (LTB4) were detected in the supernatants of tumor cells, and both showed elevated expression in cotransfected cells compared to FUBP1‐knockdown osteosarcoma cells (Figure 7E). Moreover, we blocked AA metabolic pathway using a selective PTGES inhibitor CAY10526 in *FUBP1*‐overexpressing osteosarcoma cells and stimulated it using an EP2 receptor agonist Evatanepag in *FUBP1*‐silenced osteosarcoma cells. As expected, the effect of FUBP1 overexpression on the activation of AA metabolism was hindered by the inhibitor, and the inhibitory effect of siRNAs targeting FUBP1 was stimulated by the agonist. The CCK8 assay showed that treatment with the inhibitor enhanced the effects of lobaplatin and that treatment with the agonist attenuated the effects of lobaplatin in osteosarcoma cells (Figure 7F,G). The above results indicate that FUBP1 promotes lobaplatin resistance in human osteosarcoma cells through PTGES and AA metabolism.

### 2.6 FUBP1 transcriptionally activates PTGES

**FIGURE 8:**
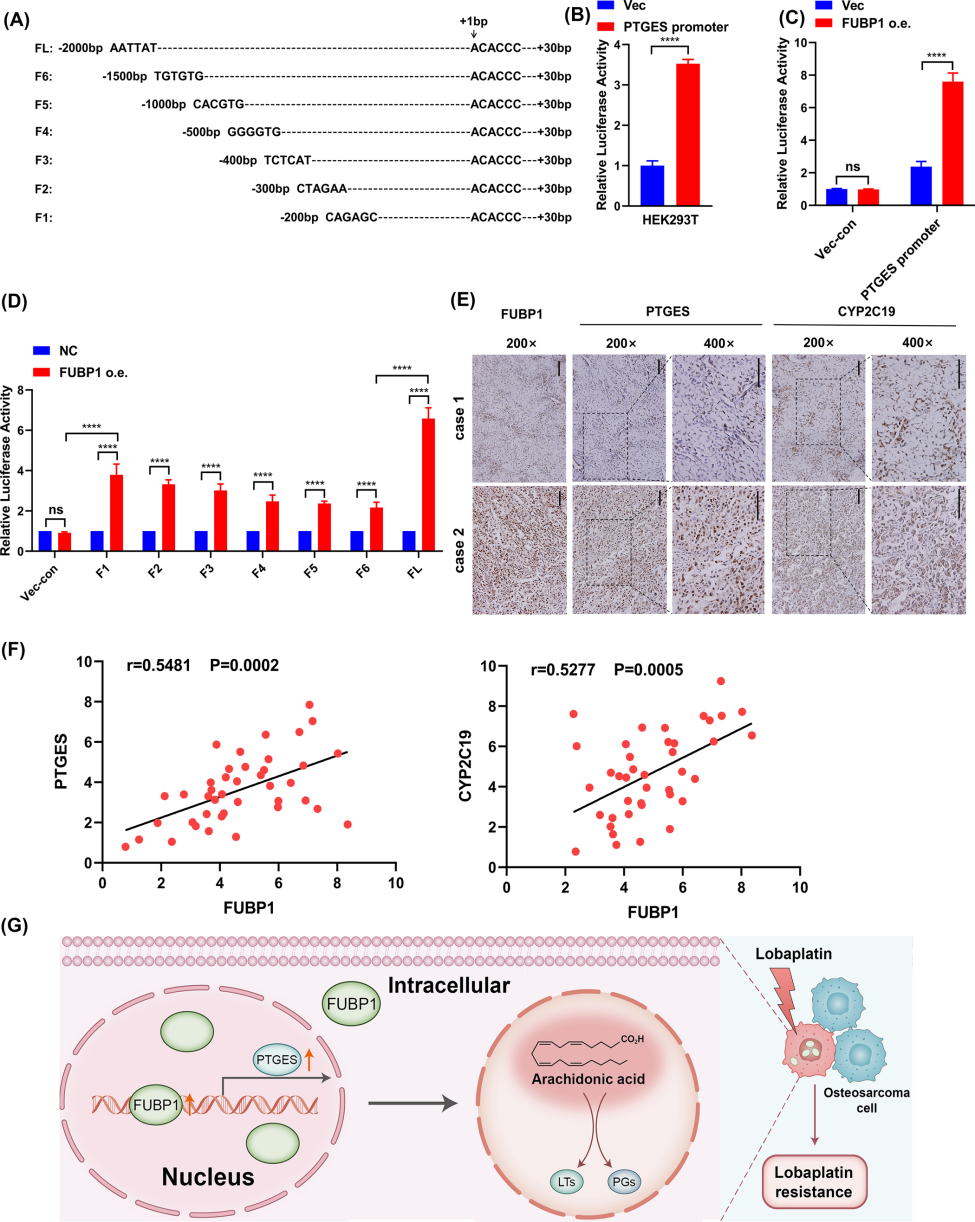
Far upstream element‐binding protein 1 (FUBP1) transcriptionally activates prostaglandin E synthase (PTGES) and correlates with PTGES and CYP2C19 in clinical osteosarcoma specimens. (A) Design of seven PTGES promoter fragments for active binding site analysis. (B) The transcriptional activity of the PTGES promoter in HEK293T cells analyzed with a luciferase reporter assay. (C) FUBP1 promotes the transcriptional activity of the PTGES promoter in HEK293T cells. Luciferase reporter plasmids driven by the PTGES promoter were transfected into FUBP1‐overexpressing or control HEK293T cells. The luciferase activity of these cells was evaluated by a dual‐luciferase reporter assay. (D) The transcriptional activities of the indicated fragments of the PTGES promoter in FUBP1‐overexpressing and control HEK293T cells were analyzed. FUBP1 may bind to −2000 to −1500 and −200 to +30 bp regions from the transcriptional start site (TSS) of the PTGES promoter. (E) The expression levels of PTGES and CYP2C19 were associated with the expression of FUBP1 in 41 primary human osteosarcoma specimens. Scale bars = 100 µm. (F) The correlation between FUBP1 and the expression of PTGES and CYP2C19 in human osteosarcoma tissue samples. (G) Hypothetical model depicting FUBP1 regulation of PTGES and activation of arachidonic acid metabolic pathway. FUBP1 serves as a therapeutic target for lobaplatin‐resistant osteosarcoma patients. ****p < 0.0001.

We next investigated the mechanism underlying FUBP1 regulation of PTGES. As FUBP1 was demonstrated to function as a transcription factor by ChIP‐seq, we speculated that FUBP1 could directly regulate PTGES at the transcriptional level. Therefore, seven PTGES promoter fragments were constructed: F1 (from −200 to 30 bp), F2 (from −300 to 30 bp), F3 (from −400 to 30 bp), F4 (from −500 to 30 bp), F5 (from −1000 to 30 bp), F6 (from −1500 to 30 bp), and FL (full length) (Figure 8A). We next examined the binding of FUBP1 to the different fragments of the PTGES promoter. As shown in Figure 8B, HEK293T cells transfected with FL PTGES promoter‐driven luciferase reporter plasmids demonstrated increased luciferase activity, displaying a well‐constructed reaction system. Next, we found that FUBP1‐overexpressing HEK293T cells transfected with FL PTGES promoter‐driven plasmids demonstrated markedly elevated luciferase activity compared to that in control cells transfected with empty vectors (Figure 8C). These results indicated that FUBP1 enhances the transcriptional activity of the PTGES promoter. Furthermore, cells transfected with fragments F1 and FL showed markedly increased luciferase activity compared to that in cells transfected with the other fragments (Figure 8D), which indicated that two potential binding sites in the PTGES promoter, upstream of 200 bp and between 1500 and 2000 bp, are responsible for the regulation of FUBP1 on the PTGES promoter.

### 2.7 Clinical relevance of FUBP1‐induced activation of PTGES and CYP2C19 in osteosarcoma

The clinical relevance of FUBP1 expression in terms of the activation of PTGES and CYP2C19 (a key CYP epoxygenase enzyme in AA metabolism) was further investigated in a group of human osteosarcoma clinical specimens using IHC analysis. We found that the expression level of FUBP1 in the clinical osteosarcoma samples was positively correlated with the expression level of PTGES and CYP2C19 (*p* = 0.002, and *p* = 0.005, Figure 8E,F). These data further support that FUBP1 upregulation promotes osteosarcoma chemoresistance by activating the AA metabolic pathway, which might lead to poor clinical outcomes for osteosarcoma patients.

## 3 DISCUSSION

In this investigation, we presented the first demonstration that FUBP1 overexpression confers lobaplatin resistance, whereas FUBP1 knockdown increases lobaplatin sensitivity in human osteosarcoma both in vitro and in vivo. Mechanistically, FUBP1 binds to the promoter of PTGES and sustains the PTGES/PGE2 axis, which then activates AA metabolism (Figure 8G). In addition, our study showed that PTGES inhibition enhances the sensitivity of osteosarcoma cells to lobaplatin, which leads to the suppression of tumor cells. Moreover, PGE2 inhibition enhances the sensitivity of osteosarcoma cells induced by FUBP1 interference. These findings reveal a novel mechanism that regulates AA metabolism in osteosarcoma and suggests a promising treatment strategy of targeting FUBP1 and AA metabolism to enhance the lobaplatin response during osteosarcoma chemoresistance.

The PTGES/PGE2 axis was found to be associated with immunosuppression, lung tumorigenesis and metastasis in a Gprc5a‐knockout mouse model.^22^ PTGES, the key enzyme for the synthesis of PGE2 in the AA pathway, was demonstrated to be essential for the tumorigenicity, migration, and metastasis of non‐small cell lung cancer (NSCLC) cells.^23^ In glioma, inhibition of mPGES‐1 blocks angiogenic Akt‐fibroblast growth factor 2/TGF‐β/vascular endothelial growth factor signaling.^24^ Weigert et al. reported that arachidonate metabolites can alter the macrophage phenotype in a dynamically changing tumor microenvironment.^25^ Blockade of the AA pathway not only decreases tumor neovascularization and growth but also reduces pulmonary metastases.^26^ More importantly, it was reported that AA could drive an adaptive response to chemotherapy‐induced stress in malignant mesothelioma, which impaired NFκB activation and affected the resistance of malignant pleural mesothelioma 3D cultures to the drug.^8^ Moreover, inhibition of mPGES‐1/PGE‐2 signaling could enhance sensitivity to cisplatin and responsiveness to gefitinib in NSCLC cells resistant to gefitinib.^27^ The AA metabolic pathway is now considered a novel preventive and therapeutic target in cancer.^28^, ^29^, ^30^ Several inhibitory natural products of metabolic enzymes have been identified with therapeutic potential against cancers. In the present study, we found that FUBP1 transcriptionally promotes the expression of PTGES and sustains the activation of the AA pathway in lobaplatin‐treatment osteosarcoma cells. Moreover, FUBP1 overexpression dramatically reduced osteosarcoma sensitivity to lobaplatin both in vitro and in vivo, whereas silencing FUBP1 enhanced osteosarcoma sensitivity. FUBP1 was found to be positively correlated with proteins relevant to AA metabolism, such as PTGES and CYP2C19, in the clinical specimens. Taken together, our results present a novel mechanism of AA pathway activation in osteosarcoma and indicate that targeting FUBP1 might be a novel therapeutic strategy for osteosarcoma patients with lobaplatin resistance.

FUBP1 is the most important member of the FBP family (which includes FUBP1, FUBP2, and FUBP3) that functions oppositely in different tumors, showing both pro‐oncogenic and tumor‐suppressive effects.^31^ FUBP1 was identified as a long tail cancer driver and widespread regulator of oncogene alternative splicing and tumor suppressor.^32^ Recently, it was reported that FUBP1 could interact with some noncoding RNAs and promote tumorigenesis in lung cancer and breast cancer by activating the proto‐oncogene MYC.^15^, ^33^ Additionally, nuclear import of FUBP1 could contribute to tumor immune evasion in cervical cancer.^34^ In osteosarcoma, we found that FUBP1 was upregulated and that overexpression of FUBP1 promoted drug resistance to lobaplatin. According to the ChIP‐seq and RNA‐seq data, FUBP1 bound to the promoter of PTGES, promoting transcription. We also found that the upregulation of PTGES significantly decreased the sensitivity of osteosarcoma cells to lobaplatin due to interference with FUBP1. Moreover, RNA‐seq showed the enrichment of the AA pathway when the sensitivity of osteosarcoma cells to lobaplatin was elevated by FUBP1 knockdown. These findings suggested that the overexpression of FUBP1 in osteosarcoma might be associated with transcriptional regulation of PTGES. Further luciferase reporter gene assays verified the regulation of FUBP1 on PTGES, and truncation assays demonstrated that −2000 to −1500 and −200 to +30 bp from the TSS were the binding regions between FUBP1 and PTGES.

Lipid metabolism plays pivotal roles in signal transduction for many cellular activities and has received remarkable attention as an emerging key effector in cancer cell behavior.^35^, ^36^ Cancer cells metabolize lipids to obtain energy and signaling molecules to proliferate, metastasize, and respond to various therapies.^37^ Seo et al. reported that the upregulation of fatty acid‐binding protein 5 induced by fatty acids drives the progression of hepatocellular carcinoma through lipid metabolism reprograming.^38^ Another study in hepatocellular carcinoma showed that acyl‐CoA synthetase long‐chain family member 4 reprograms fatty acid metabolism via c‐Myc/sterol regulatory element‐binding protein 1 signaling.^39^ Moreover, blocking cancer‐associated adipocyte (CAA) lipolysis and free fatty acid uptake has been demonstrated to have beneficial effects on tumor suppression in preclinical animal models.^40^ With respect to chemotherapy resistance, increasing evidence indicates that the hypoxic environment of tumors can trigger lipid metabolism and produce high levels of adenosine triphosphate, which is a critical factor for chemoresistance.^41^ In addition, adipocyte‐derived conditioned medium compromises tumor cell chemosensitivity to therapeutics such as carboplatin.^42^ However, personalized treatment guidance related to lipid metabolism remains to be clarified in osteosarcoma. In our study, we found that a key enzyme in the AA pathway (PTGES) was upregulated during FUBP1‐induced lobaplatin resistance. We then detected the metabolites of AA (PGE2 and LTB4), which showed increased expression in FUBP1‐overexpressing osteosarcoma cells. The selective PTGES inhibitor CAY10526 and agonist of EP2 receptor Evatanepag, which are crucial elements in AA metabolic pathway, were also used, and the chemosensitivity of osteosarcoma cells to lobaplatin was markedly reduced and elevated, respectively. These results indicate that FUBP1 can contribute to AA metabolism activation and thereby confer chemoresistance in human osteosarcoma cells.

Studies have demonstrated that the FBP family of proteins exhibits distinct functions in different cell types. For instance, FUBP2 is regarded as a potential therapeutic target against melanoma.^43^ However, FUBP3 can promote cancer progression by activating c‐Myc in colorectal, liver, and renal cancers but not prostate or bladder cancer.^44^, ^45^ Similarly, it was reported that mutation of FUBP1 might lead to lysine‐specific demethylase 1+8a deficiency and contribute to the tumorigenesis of glioma, suggesting the tumor‐suppressive effects of FUBP1,^46^ whereas competitive binding to circACTN4 could activate MYC transcription and facilitate tumor progression in breast cancer.^33^ Collectively, these findings indicate that whether FUBP1 functions as an oncoprotein or tumor suppressor depends on the tumor type. In our current study, we observed that high expression of FUBP1 was associated with poor osteosarcoma patient survival. Overexpression of FUBP1 conferred lobaplatin resistance in osteosarcoma, whereas inhibition of FUBP1 using either siRNA or lentivirus‐mediated shRNA sensitized osteosarcoma cells to lobaplatin both in vitro and in vivo. FUBP1 binds to and promotes PTGES promoter activity and sustains the AA metabolism pathway in osteosarcoma cells. Our results reveal a novel mechanism for lobaplatin resistance in osteosarcoma.

The present study has a relatively small sample size mainly because of the low incidence of osteosarcoma. It is difficult to obtain a large number of specimens. Further studies are needed to investigate the clinical value of FUBP1, PTGES, and the AA metabolism pathway in additional, larger patient cohorts. Second, in the present study, Evatanepag, an agonist of the EP2 receptor, was chosen to mediate the action of PGE2 in the AA metabolism pathway. However, the EP receptor family comprises four G protein‐coupled receptors, termed EP1 to EP4. We may also try an agonist of the EP4 receptor to mediate PGE2 in future studies.

## 4 CONCLUSION

Our findings provide evidence that FUBP1 plays a critical role in the process of lobaplatin resistance in human osteosarcoma through the transcriptional regulation of PTGES and activation of AA metabolism. Understanding the molecular mechanisms of FUBP1 in osteosarcoma progression and chemoresistance may establish FUBP1 and AA metabolism as potential therapeutic targets for the clinical treatment of osteosarcoma.

## 5 METHODS

### 5.1 Patient specimens

Clinically and histopathologically diagnosed specimens of human osteosarcoma were obtained from 60 patients who were treated with platinum‐based chemotherapy followed by tumor resection at Tangdu Hospital, Fourth Military Medical University between March 2007 and June 2021. Tumor specimens with less than 50% regression were classified as chemoresistant according to previously described criteria for rectal cancer.^47^ Clinical information for the corresponding patients was gathered. The research strategies concerning human tissues and animals were all approved by the Ethics Committee of the Fourth Military Medical University and were in accordance with the Declaration of Helsinki and the US Public Health Service Policy on Human Care and the Use of Laboratory Animals, and written informed consent was provided by the patients.

### 5.2 Cell lines and cultures

The human osteosarcoma cell lines MG63, SaOS‐2, U2OS, HOS, and SJSA‐1 were purchased from the Cell Bank of the Chinese Academy of Sciences (Shanghai, China). The osteosarcoma cell line SOSP‐9607 was established and maintained in our laboratory.^48^ MG63 and HOS cells were cultured in modified Eagle's medium, SOSP‐9607 and SJSA‐1 cells were cultured in RPMI‐1640 medium (Thermo Fisher Scientific, USA), SaOS‐2 cells were cultured in Dulbecco's modified Eagle's medium (Thermo Fisher Scientific, USA), and U2OS cells were cultured in McCoy's 5A medium with 10% fetal bovine serum (Gibco, USA). These cells were cultured at 37°C with 5% CO_2_. The human osteoblast cell line hFOB 1.19 was supplied by Jennio Biotech Co., Ltd. (Guangzhou, China) and cultured in modified Eagle's medium/F12 with 10% fetal bovine serum at 34°C.

### 5.3 Plasmids, siRNAs, and transfection

Overexpression plasmids and siRNA/shRNA oligonucleotide sequences were designed and synthesized by Shanghai GenePharma Company (Shanghai, China). For the overexpression of FUBP1, cDNA was amplified by Pfu DNA polymerase (Sangon Biotech, Shanghai, China) and inserted into the pEX‐3 (pGCMV/MCS/Neo) plasmid. The siRNA/shRNA sequences for the knockdown of FUBP1 and PTGES are shown in Table S5. Osteosarcoma cells were transfected with plasmids or siRNA/shRNA using Lipofectamine 3000 (Invitrogen, USA) according to the manufacturer's instructions. Cells were then used for assays 48–72 h post‐transfection. Overexpression and knockdown efficiency were evaluated by western blotting.

### 5.4 Lentivirus infection

Lentiviral particles and scrambled control lentiviral particles were provided by Shanghai Genechem Co., Ltd., and 1 × 10^5^ osteosarcoma cells were seeded into 6‐well culture plates and infected with lentiviral particles at 50% confluence with a multiplicity of infection of 50. The infected cells were subsequently selected in the presence of 4 µg/mL puromycin (Thermo Fisher Scientific, USA) for 7 days and maintained in medium containing 2 µg/mL puromycin to obtain stably infected cells. Real‐time q‐PCR and western blotting were performed to analyze the effect of viral infection.

### 5.5 qPCR

Total RNA was extracted using the GeneJET RNA Purification Kit (Thermo Scientific, USA), and cDNA was acquired by reverse transcription using the PrimeScript RT reagent kit (TAKARA, Japan). Quantitative real‐time PCR was performed with a TB Green Fast qPCR Mix (TAKARA, Japan). The 2^−ΔΔ^ *^Ct^* method was used to quantify the expression of each gene normalized to that of GAPDH. Detailed information on the primer sequences for each gene is shown in Table S6.

### 5.6 Western blotting analysis

Cell lysates were harvested in RIPA buffer (Cell Signaling Technology, USA) with 1% protease inhibitor cocktail (Roche, Switzerland) on ice and then quantified using a BCA protein assay kit (NCM, China). Total protein extracts (20 µg) were subjected to 10% SDS‒PAGE and then transferred to polyvinylidene difluoride membranes (Millipore, USA). The membranes were blocked with 5% nonfat milk powder (BD, USA) diluted in TBST for 2 h, incubated with primary antibodies overnight, and then incubated with HRP‐conjugated secondary antibody for 1 h (Table S7). The signal intensities of each band were quantified with Image‐Pro Plus.

### 5.7 H&E, IHC, and IF staining

Tissues were fixed with 10% formalin solution overnight at room temperature. Graded ethanol (70%, 80%, 95%, and 100%) was used for dehydration. Paraffin‐embedded tissues were cut into 3 µm slices using a microtome (LEICA, Germany). Tissue slices were deparaffinized in xylene, rehydrated in graded ethanol solutions, and stained with hematoxylin–eosin (H&E) or a specific antibody (Table S7), and the slides were observed under an upright fluorescence microscope (Olympus BX51, Japan) as previously described.^47^

### 5.8 RNA FISH

Osteosarcoma cells were seeded in six‐well plates and fixed with 4% paraformaldehyde. Cells were then treated with 0.5% Triton and prehybridized. Probes (500 nM) were incubated overnight for hybridization. RNA FISH was performed using a kit purchased from RiboBio (Guangzhou) according to the manufacturer's instructions. Cy3‐labeled FUBP1 probes (ATTCCCTCCCAGTGTGTTGTACTGC) were synthesized and provided by Servicebio Technology Co., Ltd., and images were acquired with a fluorescence microscope (Nikon ECLIPSE CI, Japan).

### 5.9 Flow cytometry

Osteosarcoma cells (3 × 10^5^) were plated in six‐well plates and incubated overnight. Cells were transfected with siRNAs or overexpression plasmids, and treatment started with lobaplatin 24 h post‐transfection. The cells were then harvested after another 24 h of incubation, washed with PBS, and resuspended in binding buffer. Cell apoptosis was evaluated using the FITC Annexin V Apoptosis Detection Kit (BD, USA) and a Coulter EPICS XL flow cytometer (Beckman‐Coulter, USA). Each sample was examined at least three times, and EXP032 ADC Analysis software was used to analyze the apoptosis data.

### 5.10 Colony formation assay

Six hundred osteosarcoma cells infected with FUBP1‐overexpressing vector, FUBP1‐knockdown vector, or their separate control vectors were seeded in 35‐mm culture dishes. Three replicates were performed for each sample. After 10–14 days of culture, the colonies were fixed with methanol for 20 min and stained with crystal violet solution (Abcam, USA) for 3 min. The colonies were dried in air and then photographed and counted.

### 5.11 Cytotoxicity assay

The sensitivity of osteosarcoma cells to lobaplatin was determined using a CCK‐8 assay. A total of 6000 cells per well were seeded in 96‐well plates for transfection with plasmids or siRNAs and then treated with different concentrations of lobaplatin. Forty‐eight hours after transfection, 10 µL of CCK‐8 reagent was added, and the cells were further incubated for another 3 h. The absorbance at 450 nm was measured using a multiscan reader (Infinite M200 PRO, Switzerland). Each experimental group contained three replicate wells. The drug resistance index was determined by calculating the half maximal inhibitory concentration (IC_50_).

### 5.12 Xenograft tumor model

Four‐week‐old female nude mice were provided by Sibeifu Company. After 1 week of adaptive feeding, the animals were categorized into four groups, including the FUBP1‐overexpression group, FUBP1‐downregulated group, and their separate vector control groups. Each group contained six animals. Animals were then subcutaneously injected with 200 µL of 1 × 10^7^/mL osteosarcoma cells into the outer flank of the left thigh. Treatment with lobaplatin (3 mg/kg body weight administered every 3 days) began when the tumor burden reached a volume of 0.125 cm^3^. Tumor volumes were calculated with the following formula by measuring the length and width every 3 days: *V* = [*W* × *L* × (*W* + *L*)/2] × 0.52.^49^ Animals were sacrificed with excessive anesthesia 6 weeks after tumor cell injection to ensure animal welfare. Tumors were obtained and weighed, and TUNEL staining assays were performed. All animal operations were in accordance with the Interdisciplinary Principles and Guidelines for the Use of Animals in Research, Testing, and Education by the New York Academy of Sciences, Ad Hoc Animal Research Committee.

### 5.13 TUNEL

TUNEL staining was performed using an In Situ Cell Death Detection Kit (Roche, Germany) according to the manufacturer's instructions. TUNEL‐positive cells were visualized and counted using the FITC channel on an upright fluorescence microscope. The proportion of TUNEL‐positive cells was calculated from at least five random fields in three wells each per group.

### 5.14 ChIP‐seq

FUBP1 ChIP‐seq was performed as previously described using 5 × 10^7^ human MG63 osteosarcoma cells.^50^ Immunoprecipitation of FUBP1 was performed with an anti‐FUBP1 antibody (Proteintech, China). ChIP‐seq datasets were analyzed using the ENCODE pipeline (https://github.com/ENCODE‐DCC/chip‐seq‐pipeline2). Motifs in the peaks were analyzed using Homer software and then aligned in the JASPAR database. Read density differences are represented as *p* values as determined by using the Mann‒Whitney‒Wilcoxon test.

### 5.15 RNA‐seq

Cells were seeded in 10‐cm culture dishes and transfected with siRNA or the negative control for FUBP1. RNA was extracted, and the integrity was assessed using the RNA nano 6000 assay kit from the Bioanalyzer 2100 system (Agilent Technologies, USA) 48 h after transfection. In total, 1 µg of RNA was used per sample for RNA sample preparation. The clustering of each sample was performed using a TruSeq PE Cluster Kit v3‐cBot‐HS (Illumina, USA) on a cBot Cluster Generation System according to the manufacturer's instructions. Differential expression between the two groups was analyzed using the DESeq2 R package (version 1.20.0). Genes with an adjusted *p* value <0.05 found by DESeq2 were considered differentially expressed. The GO annotation of the target gene sets was performed using Blast2GO. Signaling pathway analysis was performed with the KEGG database.

### 5.16 Luciferase reporter assay

A luciferase reporter assay was performed as described previously.^51^ Briefly, transient transfection was performed on 7 × 10^3^ HEK293T cells with 0.2 µg of each of the plasmids (GV238‐PTGES promoter‐wild type, GV238‐PTGES promoter‐F1, GV238‐PTGES promoter‐F2, GV238‐PTGES promoter‐F3, or GV238‐PTGES promoter‐F4, GV238‐PTGES promoter‐F5, GV238‐PTGES promoter‐F6), 0.2 µg of a FUBP1 plasmid and 0.005 µg of pRL‐TK *Renilla* plasmid using Lipofectamine 3000 Transfection Reagent (Invitrogen, USA). The absorbance (OD) values, which represent the luciferase and Renilla signals, were detected at 48 h post‐transfection using the Dual‐Luciferase Reporter Assay Kit (Promega, USA) according to the manufacturer's instructions.

### 5.17 Detection of PGE2 and LTB4 in cell media

The PGE2 and LTB4 ELISA Kit (Cayman Chemical, USA) were used to semiquantitatively determine the amount of the above two classical metabolites of AA after various treatments according to the manufacturer's instructions. The absorbance at 420 nm is inversely proportional to the amount of PGE2 and LTB4 contained in the sample or standard.

### 5.18 Statistical analysis

Statistical analysis was performed with GraphPad Prism 7 software (GraphPad, RRID: SCR_002798) and SPSS 25.0 (IBM, RRID: SCR_002865). All experiments were carried out at least three times, and the mean ± standard error of the mean was calculated for each independent experiment. Comparisons between two groups were analyzed using Student's *t* test, and differences among multiple groups were analyzed using one‐way or two‐way ANOVA. IC_50_ values were analyzed by regression analysis. The false discovery rate was adjusted using Benjamini and Hochberg's approach. *p* Values less than 0.05 were considered significant.

## AUTHOR CONTRIBUTIONS

Wei Zhang, Li Gong, and Zheng Guo contributed to the design of the study; Qiong Ma, Jin Sun, Chengpei Zhou, and Chenyu Li performed data acquisition; Qiong Ma, Jin Sun, and Huan Wang performed data analysis and interpretation; Yonghong Wu, Yanhua Wen, and Xingguang Ren performed clinical data collection and sample disposal; Qiong Ma drafted the manuscript; Xiaoyu Zhang and Li Gong performed manuscript revision. All authors approved the final version of the manuscript.

## CONFLICT OF INTEREST STATEMENT

The authors declare no conflict of interests.

## ETHICS STATEMENT

The research strategies concerning human tissues and animals were all approved by the Ethics Committee of the Fourth Military Medical University (No. TDLL‐202205‐07, No. IACUC‐20220663) and were in accordance with the Declaration of Helsinki and the US Public Health Service Policy on Human Care and the Use of Laboratory Animals, and written informed consent was provided by the patients.

## Supporting information

Supporting Information

## Data Availability

The datasets analyzed in this study are available upon reasonable request from the corresponding author, and RNA sequencing data have been deposited to GEO platform (https://www.ncbi.nlm.nih.gov/geo) and are available under accession number: GSE210740.

## References

[1] Corre I , Verrecchia F , Crenn V , Redini F , Trichet V . The osteosarcoma microenvironment: a complex but targetable ecosystem. Cells. 2020;9:976. 32326444 10.3390/cells9040976 PMC7226971

[2] Chen C , Xie L , Ren T , Huang Y , Xu J , Guo W . Immunotherapy for osteosarcoma: fundamental mechanism, rationale, and recent breakthroughs. Cancer Lett. 2021;500:1‐10. 33359211 10.1016/j.canlet.2020.12.024

[3] Zhang R , Wang H , Li E , et al. Quantitative phosphoproteomic analysis reveals chemoresistance‐related proteins and signaling pathways induced by rhIL‐6 in human osteosarcoma cells. Cancer Cell Int. 2021;21:581. 34717622 10.1186/s12935-021-02286-z PMC8557500

[4] Oude Munnink T , van der Meer A , de Haan J , Touw D , van Kruchten M . Reversible impaired methotrexate clearance after platinum‐based chemotherapy for osteosarcoma. Ther Drug Monit. 2019;41:693‐695. 31169759 10.1097/FTD.0000000000000662

[5] Martin SA , Brash AR , Murphy RC . The discovery and early structural studies of arachidonic acid. J Lipid Res. 2016;57:1126‐1132. 27142391 10.1194/jlr.R068072 PMC4918860

[6] Hanna VS , Hafez EAA . Synopsis of arachidonic acid metabolism: a review. J Adv Res. 2018;11:23‐32. 30034873 10.1016/j.jare.2018.03.005 PMC6052663

[7] Lee JY , Nam M , Son HY , et al. Polyunsaturated fatty acid biosynthesis pathway determines ferroptosis sensitivity in gastric cancer. Proc Natl Acad Sci USA. 2020;117:32433‐32442. 33288688 10.1073/pnas.2006828117 PMC7768719

[8] Cioce M , Canino C , Pass H , Blandino G , Strano S , Fazio VM . Arachidonic acid drives adaptive responses to chemotherapy‐induced stress in malignant mesothelioma. J Exp Clin Cancer Res. 2021;40:344. 34727953 10.1186/s13046-021-02118-y PMC8561918

[9] Zhao Y , Cui L , Pan Y , et al. Berberine inhibits the chemotherapy‐induced repopulation by suppressing the arachidonic acid metabolic pathway and phosphorylation of FAK in ovarian cancer. Cell Prolif. 2017;50:e12393. 28990249 10.1111/cpr.12393 PMC6529084

[10] Hirata H , Sugimachi K , Komatsu H , et al. Decreased expression of fructose‐1,6‐bisphosphatase associates with glucose metabolism and tumor progression in hepatocellular carcinoma. Cancer Res. 2016;76:3265‐3276. 27197151 10.1158/0008-5472.CAN-15-2601

[11] Venturutti L , Cordo Russo RI , Rivas MA , et al. MiR‐16 mediates trastuzumab and lapatinib response in ErbB‐2‐positive breast and gastric cancer via its novel targets CCNJ and FUBP1. Oncogene. 2016;35:6189‐6202. 27157613 10.1038/onc.2016.151 PMC5832962

[12] Jiang P , Huang M , Qi W , et al. FUBP1 promotes neuroblastoma proliferation via enhancing glycolysis‐a new possible marker of malignancy for neuroblastoma. J Exp Clin Cancer Res. 2019;38:400. 31511046 10.1186/s13046-019-1414-6 PMC6737630

[13] Hoang VT , Verma D , Godavarthy PS , et al. The transcriptional regulator FUBP1 influences disease outcome in murine and human myeloid leukemia. Leukemia. 2019;33:1700‐1712. 30635626 10.1038/s41375-018-0358-8

[14] Zhong Q , Liu ZH , Lin ZR , et al. The RARS‐MAD1L1 fusion gene induces cancer stem cell‐like properties and therapeutic resistance in nasopharyngeal carcinoma. Clin Cancer Res. 2018;24:659‐673. 29133573 10.1158/1078-0432.CCR-17-0352 PMC5796860

[15] Qian X , Yang J , Qiu Q , et al. LCAT3, a novel m6A‐regulated long non‐coding RNA, plays an oncogenic role in lung cancer via binding with FUBP1 to activate c‐MYC. J Hematol Oncol. 2021;14:112. 34274028 10.1186/s13045-021-01123-0 PMC8285886

[16] Zheng Y , Dubois W , Benham C , Batchelor E , Levens D . FUBP1 and FUBP2 enforce distinct epigenetic setpoints for MYC expression in primary single murine cells. Commun Biol. 2020;3:545. 33005010 10.1038/s42003-020-01264-x PMC7530719

[17] Liu W , Xiong X , Chen W , et al. High expression of FUSE binding protein 1 in breast cancer stimulates cell proliferation and diminishes drug sensitivity. Int J Oncol. 2020;57:488‐499. 32626933 10.3892/ijo.2020.5080 PMC7307591

[18] Wang B , Fan P , Zhao J , Wu H , Jin X , Wu H . FBP1 loss contributes to BET inhibitors resistance by undermining c‐Myc expression in pancreatic ductal adenocarcinoma. J Exp Clin Cancer Res. 2018;37:224. 30201002 10.1186/s13046-018-0888-y PMC6131902

[19] Wang H , Li B , Yan K , et al. Protein and signaling pathway responses to rhIL‐6 intervention before lobaplatin treatment in osteosarcoma cells. Front Oncol. 2021;11:602712. 33791202 10.3389/fonc.2021.602712 PMC8006349

[20] Zhang Y , Yao Y , Luo J , et al. Microsomal prostaglandin E2 synthase‐1 and its inhibitors: molecular mechanisms and therapeutic significance. Pharmacol Res. 2022;175:105977. 34798265 10.1016/j.phrs.2021.105977

[21] Korotkova M , Jakobsson PJ . Characterization of microsomal prostaglandin E synthase 1 inhibitors. Basic Clin Pharmacol Toxicol. 2014;114:64‐69. 24138533 10.1111/bcpt.12162

[22] Wang T , Jing B , Xu D , et al. PTGES/PGE2 signaling links immunosuppression and lung metastasis in Gprc5a‐knockout mouse model. Oncogene. 2020;39:3179‐3194. 32060421 10.1038/s41388-020-1207-6 PMC7142021

[23] Wang T , Jing B , Sun B , et al. Stabilization of PTGES by deubiquitinase USP9X promotes metastatic features of lung cancer via PGE2 signaling. Am J Cancer Res. 2019;9:1145‐1160. 31285948 PMC6610053

[24] Wang C , Chen Y , Wang Y , et al. Inhibition of COX‐2, mPGES‐1 and CYP4A by isoliquiritigenin blocks the angiogenic Akt signaling in glioma through ceRNA effect of miR‐194‐5p and lncRNA NEAT1. J Exp Clin Cancer Res. 2019;38:371. 31438982 10.1186/s13046-019-1361-2 PMC6704644

[25] Weigert A , Strack E , Snodgrass RG , Brune B . mPGES‐1 and ALOX5/‐15 in tumor‐associated macrophages. Cancer Metastasis Rev. 2018;37:317‐334. 29808459 10.1007/s10555-018-9731-3

[26] Borin TF , Angara K , Rashid MH , Achyut BR , Arbab AS . Arachidonic acid metabolite as a novel therapeutic target in breast cancer metastasis. Int J Mol Sci. 2017;18:2661. 29292756 10.3390/ijms18122661 PMC5751263

[27] Terzuoli E , Costanza F , Ciccone V , Ziche M , Morbidelli L , Donnini S . mPGES‐1 as a new target to overcome acquired resistance to gefitinib in non‐small cell lung cancer cell lines. Prostaglandins Other Lipid Mediat. 2019;143:106344. 31207300 10.1016/j.prostaglandins.2019.106344

[28] Yarla NS , Bishayee A , Sethi G , et al. Targeting arachidonic acid pathway by natural products for cancer prevention and therapy. Semin Cancer Biol. 2016;40‐41:48‐81. 10.1016/j.semcancer.2016.02.001 26853158

[29] Dietze R , Hammoud MK , Gomez‐Serrano M , et al. Phosphoproteomics identify arachidonic‐acid‐regulated signal transduction pathways modulating macrophage functions with implications for ovarian cancer. Theranostics. 2021;11:1377‐1395. 33391540 10.7150/thno.52442 PMC7738879

[30] Denizot Y , Najid A , Rigaud M . Incorporation of arachidonic acid in a human cancer gastric tumor cell line (HGT) at various stages of cell proliferation. Cancer Lett. 1993;68:199‐205. 8443793 10.1016/0304-3835(93)90147-2

[31] Zhou W , Chung YJ , Parrilla Castellar ER , et al. Far upstream element binding protein plays a crucial role in embryonic development, hematopoiesis, and stabilizing myc expression levels. Am J Pathol. 2016;186:701‐715. 26774856 10.1016/j.ajpath.2015.10.028 PMC4816710

[32] Elman JS , Ni TK , Mengwasser KE , et al. Identification of FUBP1 as a long tail cancer driver and widespread regulator of tumor suppressor and oncogene alternative splicing. Cell Rep. 2019;28:3435‐3449.e5. 31553912 10.1016/j.celrep.2019.08.060 PMC7297508

[33] Wang X , Xing L , Yang R , et al. The circACTN4 interacts with FUBP1 to promote tumorigenesis and progression of breast cancer by regulating the expression of proto‐oncogene MYC. Mol Cancer. 2021;20:91. 34116677 10.1186/s12943-021-01383-x PMC8194204

[34] Yang B , Chen J , Teng Y . TNPO1‐mediated nuclear import of FUBP1 contributes to tumor immune evasion by increasing NRP1 expression in cervical cancer. J Immunol Res. 2021;2021:9994004. 33987449 10.1155/2021/9994004 PMC8093035

[35] Butler LM , Perone Y , Dehairs J , et al. Lipids and cancer: emerging roles in pathogenesis, diagnosis and therapeutic intervention. Adv Drug Deliv Rev. 2020;159:245‐293. 32711004 10.1016/j.addr.2020.07.013 PMC7736102

[36] Begicevic RR , Arfuso F , Falasca M . Bioactive lipids in cancer stem cells. World J Stem Cells. 2019;11:693‐704. 31616544 10.4252/wjsc.v11.i9.693 PMC6789187

[37] Bian X , Liu R , Meng Y , Xing D , Xu D , Lu Z . Lipid metabolism and cancer. J Exp Med. 2021;218:e20201606. 33601415 10.1084/jem.20201606 PMC7754673

[38] Seo J , Jeong DW , Park JW , Lee KW , Fukuda J , Chun YS . Fatty‐acid‐induced FABP5/HIF‐1 reprograms lipid metabolism and enhances the proliferation of liver cancer cells. Commun Biol. 2020;3:638. 33128030 10.1038/s42003-020-01367-5 PMC7599230

[39] Chen J , Ding C , Chen Y , et al. ACSL4 reprograms fatty acid metabolism in hepatocellular carcinoma via c‐Myc/SREBP1 pathway. Cancer Lett. 2021;502:154‐165. 33340617 10.1016/j.canlet.2020.12.019

[40] Cao Y . Adipocyte and lipid metabolism in cancer drug resistance. J Clin Invest. 2019;129:3006‐3017. 31264969 10.1172/JCI127201 PMC6668696

[41] Mukherjee A , Chiang CY , Daifotis HA , et al. Adipocyte‐induced FABP4 expression in ovarian cancer cells promotes metastasis and mediates carboplatin resistance. Cancer Res. 2020;80:1748‐1761. 32054768 10.1158/0008-5472.CAN-19-1999 PMC10656748

[42] Bao K , Li Y , Wei J , et al. Fangchinoline suppresses conjunctival melanoma by directly binding FUBP2 and inhibiting the homologous recombination pathway. Cell Death Dis. 2021;12:380. 33828201 10.1038/s41419-021-03653-4 PMC8027391

[43] Gao Q , Zhou R , Meng Y , et al. Long noncoding RNA CMPK2 promotes colorectal cancer progression by activating the FUBP3‐c‐Myc axis. Oncogene. 2020;39:3926‐3938. 32203166 10.1038/s41388-020-1266-8

[44] Brauckhoff A , Malz M , Tschaharganeh D , et al. Nuclear expression of the ubiquitin ligase seven in absentia homolog (SIAH)‐1 induces proliferation and migration of liver cancer cells. J Hepatol. 2011;55:1049‐1057. 21356256 10.1016/j.jhep.2011.02.019

[45] Weber A , Kristiansen I , Johannsen M , et al. The FUSE binding proteins FBP1 and FBP3 are potential c‐Myc regulators in renal, but not in prostate and bladder cancer. BMC Cancer. 2008;8:369. 19087307 10.1186/1471-2407-8-369 PMC2631590

[46] Hwang I , Cao D , Na Y , et al. Far upstream element‐binding protein 1 regulates LSD1 alternative splicing to promote terminal differentiation of neural progenitors. Stem Cell Rep. 2018;10:1208‐1221. 10.1016/j.stemcr.2018.02.013 PMC5998560 29606613

[47] Rodel C , Martus P , Papadoupolos T , et al. Prognostic significance of tumor regression after preoperative chemoradiotherapy for rectal cancer. J Clin Oncol. 2005;23:8688‐8696. 16246976 10.1200/JCO.2005.02.1329

[48] Chen X , Yang TT , Wang W , et al. Establishment and characterization of human osteosarcoma cell lines with different pulmonary metastatic potentials. Cytotechnology. 2009;61:37‐44. 20016965 10.1007/s10616-009-9239-3 PMC2795143

[49] Rocha GZ , Dias MM , Ropelle ER , et al. Metformin amplifies chemotherapy‐induced AMPK activation and antitumoral growth. Clin Cancer Res. 2011;17:3993‐4005. 21543517 10.1158/1078-0432.CCR-10-2243

[50] Milewski D , Shukla S , Gryder BE , et al. FOXF1 is required for the oncogenic properties of PAX3‐FOXO1 in rhabdomyosarcoma. Oncogene. 2021;40:2182‐2199. 33627785 10.1038/s41388-021-01694-9 PMC8005492

[51] Gong L , Song J , Lin X , et al. Serine‐arginine protein kinase 1 promotes a cancer stem cell‐like phenotype through activation of Wnt/beta‐catenin signalling in NSCLC. J Pathol. 2016;240:184‐196. 27391422 10.1002/path.4767

