# Supplementary material for: Far upstream element‐binding protein 1 confers lobaplatin resistance by transcriptionally activating PTGES and facilitating the arachidonic acid metabolic pathway in osteosarcoma
Source: MedComm (2020). 2023 May 9;4(3):e257. doi: 10.1002/mco2.257 (PMC10170244; doi:10.1002/mco2.257)
Supplement: Supplementary file 1 — Supporting Information [file MCO2-4-e257-s001.docx]

**Far upstream element-binding protein 1 confers lobaplatin resistance by transcriptionally activating PTGES and facilitating the arachidonic acid metabolic pathway in osteosarcoma**

**Running title:** **FUBP1 confers lobaplatin resistance in OS**

Qiong Ma^1,2#^, Jin Sun^2#^, Huan Wang^2#^, Chengpei Zhou^2^, Chenyu Li^2^, Yonghong Wu^2^, Yanhua Wen^2^, Xiaoyu Zhang^2^, Xingguang Ren^2^, Zheng Guo^2*^, Li Gong^1*^, Wei Zhang^1*^

^1^Department of Pathology, Tangdu Hospital, Air Force Medical University, 569 Xinsi Road, Xi’an 710038, China

^2^Orthopedic Oncology Institute, Department of Orthopedic Surgery, Tangdu Hospital, Air Force Medical University, 569 Xinsi Road, Xi’an 710038, China

***Corresponding authors**

Wei Zhang:

[zhwlyh@fmmu.edu.cn](mailto:zhwlyh@fmmu.edu.cn);

Li Gong:

[glzwd16@fmmu.edu.cn](mailto:glzwd16@fmmu.edu.cn);

Zheng Guo:

[guozheng@fmmu.edu.cn](mailto:guozheng@fmmu.edu.cn)

**^#^Qiong Ma, Jin Sun, Huan Wang contributed equally.**

**Supplementary Tables**

**Table S1. Clinical pathological features and demographics of osteosarcoma patient cohorts**

| **Gender** | **Age** | **Clinical**  **Diagnosis** | **Pathological**  **Diagnosis** | **Staging** | **Distant Metastasis** | **Die or Survive** |
| --- | --- | --- | --- | --- | --- | --- |
| Male | 17 | Left distal femur mass | Osteoblastic OS | ⅡB | No | die |
| Male | 18 | Left proximal tibia tumor | Common type OS | ⅡA | No | Survive |
| Male | 12 | Left distal femur OS combined with pathological fracture | OS (malignant fibr ous histocytoma) | ⅡA | No | die |
| Female | 10 | Left distal femur OS | Osteoblastic OS | ⅡB | No | die |
| Male | 18 | Left distal femur mass | Common type OS | ⅡB | No | die |
| Female | 21 | Right distal femur OS | Common type OS | ⅡA | No | Survive |
| Male | 15 | Left lower femur mass | OS（sclerotic type） | ⅡB | No | Survive |
| Male | 15 | Left distal tibia OS | Common type OS | ⅡB | No | Die |
| Male | 16 | Left lower femur malignant tumor | Common type OS | ⅡB | No | Survive |
| Female | 14 | Right distal radius mass | common type OS | ⅡA | Pulmonary metastasis | Survive |
| Male | 15 | Right radius OS | Osteoblastic OS | ⅡA | Pulmonary metastasis | Survive with amputation |
| Female | 26 | Left proximal  tibia OS | OS (Invasion of popliteal fossa) | ⅡA | No | Survive |
| Male | 19 | Recurrent right pelvic OS | Small cell OS  with thrombus | ⅡA | No | Die |
| Female | 14 | Left distal femur OS | Talangiectatic OS | ⅡB | No | Survive |
| Female | 37 | Right distal femur OS | Common type OS | ⅡB | No | Survive |
| Male | 15 | Right distal femur OS | Common type OS | ⅡB | No | Die |
| Male | 15 | Recurrent left distal femur mass | Common type OS | ⅡB | No | Die |
| Female | 12 | Right proximal humerus mass | Common type OS | ⅡB | No | Survive |
| Female | 13 | Right proximal humerus OS | Common type OS with thrombus | Ⅲ | Pulmonary metastasis | Die |
| Female | 14 | Left iliac lesions | Small cell OS | ⅡB | No | Die |
| **Gender** | **Age** | **Clinical**  **Diagnosis** | **Pathological**  **Diagnosis** | **Staging** | **Distant Metastasis** | **Die or Survive** |
| Male | 13 | Right lower femur mass | Osteoblastic OS | ⅡB | No | Die |
| Male | 13 | Right upper tibia mass | Common type OS with necrosis | Ⅲ | Pelvic metastasis | Die |
| Male | 40 | Left distal femur mass | Talangiectatic OS | ⅡA | No | Die |
| Female | 43 | Recurrent left iliac OS | Osteoblastic OS | Ⅲ | Pulmanary and lumbar metastasis | Survive |
| Female | 14 | Recurrent left lower femur OS | Common type OS | Ⅲ | Pulmanary metastasis | Die |
| Male | 11 | Left distal femur OS | Common type OS | ⅡB | Left humeral metastasis | Die |
| Male | 18 | Right lower femur mass | OS (malignant fibrous histocytoma) | ⅡB | No | Die |
| Male | 22 | Right distal femur mass | Fibroblastic OS | ⅡB | No | Survive |
| Male | 9 | Left distal femur mass | Common type OS | ⅡB | No | Survive |
| Female | 12 | Left proximal femur mass | Common type OS | ⅡB | No | Survive |
| Female | 14 | Right distal femur mass | Small cell OS | ⅡB | No | Survive |
| Female | 12 | Right lower femur OS | Common type OS | ⅡB | No | Survive with amputation |
| Male | 12 | Left lower femur OS | Common type OS | ⅡB | No | Die |
| Male | 19 | Left distal femur OS | Common type OS | ⅡB | No | Survive |
| Male | 14 | Right distal tibia OS | Common type OS | ⅡB | No | Die |
| Female | 15 | Recurrent left lower femur OS | Common type OS | ⅡB | No | Die |
| Male | 22 | Right distal femur OS | Common type OS | ⅡB | No | Survive |
| Male | 12 | Left lower femur mass | Fibroblastic OS | ⅡB | No | Die |
| Female | 8 | Left distal femur mass | Talangiectatic OS | Ⅲ | Subcutaneous  metastasis | Die |
| Female | 14 | Left lower femur mass | Common type OS | ⅡB | No | Survive |
| Male | 17 | Right proximal tibia OS | Common type OS | ⅡA | No | Survive |
| Male | 22 | Left femur OS | Common type OS | ⅡB | No | Survive |
| Female | 11 | Right proximal humerus OS | Small cell OS | ⅡB | No | Die |
| **Gender** | **Age** | **Clinical**  **Diagnosis** | **Pathological**  **Diagnosis** | **Staging** | **Distant Metastasis** | **Die or Survive** |
| Male | 17 | Left proximal tibia OS | Fibroblastic OS | ⅡB | No | Die |
| Female | 14 | Left femur OS | Common type OS | Ⅲ | Left humeral metastasis | Die |
| Male | 16 | Left proximal tibia OS | Common type OS | ⅡB | No | Survive |
| Female | 25 | Right fibular mass | Common type OS | Ⅲ | Pulmonary metastasis | Die |
| Male | 15 | Left proximal tibia mass | Osteoblastic OS | ⅡB | No | Survive |
| Male | 14 | Left distal femur mass | Common type OS | ⅡB | No | Die |
| Female | 12 | Right distal femur OS | Osteoblastic OS | ⅡA | No | Survive |
| Female | 68 | Right distal femur mass | Osteoblastic OS | ⅡA | No | Survive |
| Male | 15 | Left distal femur OS | Common type OS | ⅡA | No | Survive |
| Male | 17 | Left proximal tibia OS | Chondroblastic OS | ⅡB | No | Die |
| Male | 11 | Right middle and lower femur OS | Common type OS | ⅡB | No | Die |
| Male | 15 | Right middle and lower femur mass | Common type OS | Ⅲ | Pulmonary metastasis | Die |
| Female | 16 | Left proximal tibia OS | Common type OS | ⅡA | No | Survive |
| Female | 14 | Left middle femur OS | Talangiectatic OS | Ⅲ | Pulmonary metastasis | Survive |
| Male | 10 | Left proximal humerus OS | Fibroblastic OS | ⅡB | No | Die |
| Male | 18 | Right proximal tibia OS | Fibroblastic OS | ⅡA | No | Survive |
| Male | 19 | Right distal femur OS | Common type OS | ⅡB | No | Survive with amputation |
| Femal | 19 | Left proximal femur parosteal OS | Parosteal OS | ⅡA | No | Survive |

OS: osteosarcoma

**Table S2. Candidate intersection gene list of ChIP-seq and RNA-seq**

| **Gene ID** | **Gene Name** | **Log_2_ Fold Change** | **P value** | **Up/Down** | **Gene description** |
| --- | --- | --- | --- | --- | --- |
| ENSG00000198353 | HOXC4 | 3.045 | 2.863E-30 | Down | homeobox C4 |
| ENSG00000196562 | SULF2 | 2.680 | 2.835E-24 | Down | sulfatase 2 |
| ENSG00000148344 | PTGES | 2.678 | 1.335E-231 | Down | prostaglandin E synthase |
| ENSG00000259207 | ITGB3 | 2.058 | 0.000106 | Down | integrin subunit beta 3 |
| ENSG00000148180 | GSN | 1.891 | 2.687E-72 | Down | gelsolin |
| ENSG00000116701 | NCF2 | 1.690 | 0.0000563 | Down | neutrophil cytosolic factor 2 |
| ENSG00000146674 | IGFBP3 | 2.076 | 2.344E-20 | UP | insulin like growth factor binding protein 3 |
| ENSG00000173599 | PC | 1.109 | 2.360E-45 | Down | pyruvate carboxylase |

**Table S3. Information of GO enrichment analysis**

| **Category** | **Term ID** | **Term description** | | **PP value** |
| --- | --- | --- | --- | --- |
| BP (Biological Process) | GO:0043062 | extracellular structure organization | 3.53E-07 | |
|  | GO:0030198 | extracellular matrix organization | 1.01E-06 | |
|  | GO:0010469 | regulation of signaling receptor activity | 5.85E-06 | |
|  | **GO:0033559** | **unsaturated fatty acid metabolic process** | **3.38 E-05** | |
|  | **GO:0006636** | **unsaturated fatty acid biosynthetic process** | **3.43 E-05** | |
| CC (Cellular Component) | GO:0031012 | extracellular matrix | 1.24E-07 | |
|  | GO:0005578 | proteinaceous extracellular matrix | 6.42E-06 | |
|  | GO:0005788 | endoplasmic reticulum lumen | 5.43 E-05 | |
| MF (Molecular Function) | GO:0005520 | insulin-like growth factor binding | 1.34E-06 | |
|  | GO:0019838 | growth factor binding | 2.35E-06 | |
|  | GO:0048018 | receptor ligand activity | 4.93E-06 | |
|  | GO:0030545 | receptor regulator activity | 5.04 E-06 | |
|  | GO:0019955 | cytokine binding | 4.18 E-05 | |
|  | GO:0004896 | cytokine receptor activity | 4.98 E-05 | |
|  | GO:0001968 | fibronectin binding | 5.75 E-05 | |
|  | GO:0050840 | extracellular matrix binding | 8.23 E-05 | |
|  | GO:0005125 | cytokine activity | 0.00015 | |
|  | GO:0001664 | G-protein coupled receptor binding | 0.00039 | |

**Table S4. Information of KEGG enrichment analysis**

| **KEGG ID** | **Term description** | **P value** |
| --- | --- | --- |
| hsa04060 | Cytokine-cytokine receptor interaction | 2.09E-05 |
| hsa04115 | p53 signaling pathway | 4.58E-05 |
| hsa04151  hsa05165  hsa04974  hsa04512  hsa04721 | PI3K-Akt signaling pathway  Human papillomavirus infection  Protein digestion and absorption  ECM-receptor interaction  Synaptic vesicle cycle | 5.65E-05  0.00033  0.0018  0.0021  0.0035 |
| hsa04934 | Cushing syndrome | 0.0055 |
| hsa04514 | Cell adhesion molecules (CAMs) | 0.0068 |
| hsa04630 | JAK-STAT signaling pathway | 0.0075 |
| hsa04080 | Neuroactive ligand-receptor interaction | 0.0076 |
| hsa05162 | Measles | 0.0097 |
| hsa05323 | Rheumatoid arthritis | 0.011 |
| hsa04924 | Renin secretion | 0.011 |
| hsa04550 | Signaling pathways regulating pluripotency of stem cells | 0.012 |
| **hsa00590** | **Arachidonic acid metabolism** | **0.012** |
| hsa05163 | Human cytomegalovirus infection | 0.019 |
| hsa04145 | Phagosome | 0.020 |
| hsa05200 | Pathways in cancer | 0.022 |
| hsa04142 | Lysosome | 0.029 |
| hsa05169 | Epstein-Barr virus infection | 0.031 |
| hsa04390 | Hippo signaling pathway | 0.031 |
| hsa03320 | PPAR signaling pathway | 0.033 |
| hsa04727 | GABAergic synapse | 0.033 |
| hsa04657 | IL-17 signaling pathway | 0.034 |
| hsa05217 | Basal cell carcinoma | 0.037 |
| hsa05219 | Bladder cancer | 0.038 |
| hsa04350 | TGF-beta signaling pathway | 0.038 |
| hsa04926 | Relaxin signaling pathway | 0.039 |
| hsa04261 | Adrenergic signaling in cardiomyocytes | 0.042 |
| hsa04724 | Glutamatergic synapse | 0.045 |
| hsa04950 | Maturity onset diabetes of the young | 0.047 |

**Table S5. siRNA and shRNA sequences of *FUBP1* and *PTGES***

| ***FUBP1*** | **RNAi#1** | 5′ -GGUGUUCGC AUUCAGUUUATT-3′ (sense) |
| --- | --- | --- |
|  |  | 5′ -UAAAC UGAAUGCGAACACCTT-3′ (antisense) |
|  | **RNAi#2** | 5′ -GGUGCUGACAAACCUCUUATT-3′ (sense) |
|  |  | 5′ -UAA GAGGU UUGUCAGCACCTT-3′ (antisense) |
|  | **RNAi#3** | 5′ -CGGCA ACUCAUAGAAGAAATT-3′ (sense) |
|  |  | 5′ -UUUCUUCUAUG AGUUGCCGTT-3′ (antisense) |
|  | **shRNA#1** | 5′ -GGUGUUCGC AUUCAGUUUATT-3′ (sense) |
|  |  | 5′ -UAAAC UGAAUGCGAACACCTT-3′ (antisense) |
|  | **shRNA#2** | 5′ -GGUGCUGACAAACCUCUUATT-3′ (sense) |
|  |  | 5′ -UAA GAGGU UUGUCAGCACCTT-3′ (antisense) |
| ***PTGES*** | **RNAi#1** | 5′ -GCUUCGUCUACUCCUUUCUTT-3′ (sense) |
|  |  | 5′ -AGAAAGGAGUAGACGAAGCTT-3′ (antisense) |
|  | **RNAi#2** | 5′ -CUGCUGGUCAUCAAGAUGUTT-3′ (sense) |
|  |  | 5′ -ACAUCUUGAUGACCAGCAGTT-3′ (antisense) |

**Table S6. Primers used in the study**

| **Real-Time Quantitative Reverse Transcription PCR** | |
| --- | --- |
| **FUBP1** | F: 5’-CAACCAGATGCTAAGAAAGTTGC-3’ |
|  | R: 5’-CCTCCTCTGCCAATTATGAATCC-3’ |
| **IGFBP3** | F: 5’-GCCAGCTCCAGGAAATGCTA-3’ |
|  | R: 5’-GGGGTGGAACTTGGGATCAG -3’ |
| **PC** | F: 5’-GACGGCGAGGAGATAGTGT-3’ |
|  | R: 5’-TGGCAATCTCACCTCTGTTGG-3’ |
| **PTGES** | F: 5’-CCCAAGGTTTGAGTCCCTCC-3’ |
|  | R: 5’-CCCATCAAGGGGACATTTGC-3’ |
| **GSN** | F: 5’-GCACGGAGAGGAACCAAGAA-3’ |
|  | F: 5’-CGGCAAGTCATTCCAGGTCT-3’ |
| **ITGB3** | F: 5’-ACCAGTAACCTGCGGATTGG-3’ |
|  | F: 5’-CTCATTGAAGCGGGTCACCT-3’ |
| **NCF2** | F: 5’-CAGGTCATGCCAGGGAACAT-3’ |
|  | F: 5’-GGAACTAGGAGGAGCTGGGA-3’ |
| **HOXC4** | F: 5’-TCTCTTCCTTCCCgACCCTC-3’ |
|  | F: 5’-AGGAAGAGCCTGGGAGAGAG-3’ |
| **SULF2** | F: 5’-TCGAAACATGGACCTGGATGG-3’ |
|  | F: 5’-TCATTGCCTGTGCAGTCAGG-3’ |
| **GAPDH** | F: 5’-CTCCTCCACCTTTGACGCTG-3’ |
|  | R: 5’-TCCTCTTGTGCTCTTGCTGG-3’ |

**Table S7. Antibodies and chemicals used in the study**

| Antibody | Catalogue | Producer | Application |
| --- | --- | --- | --- |
| FUBP1(1) | Ab181111 | Abcam | WB, 1:2000  IHC, 1:500  IF, 1:250 |
| FUBP1(2) | 24864-1-AP | Proteintech | ChIP, 1:200 |
| Histone H3 | Ab4729 | Abcam | 2 µg for 25 µg of chromatin |
| cleaved-PARP | 94885 | Cell Signaling | WB, 1:1000 |
| cleaved-caspase 9 | 20750 | Cell Signaling | WB, 1:1000 |
| cleaved-caspase 3 | AF7022 | Affinity | WB, 1:500  IHC, 1:50 |
| PTGES | 160140 | Cayman chemicals | WB, 1:200  IHC, 1:50 |
| Ki-67 | 9449T | Cell Signaling | IHC, 1:200 |
| CYP2C19 | ab137015 | Abcam | IHC, 1:100 |
| GAPDH | 60004-1-Ig | Proteintech | WB, 1:20000 |
| α-Tubulin | 66031-1-Ig | Proteintech | WB, 1:20000 |
| β-actin | 81115-1-RR | Proteintech | WB, 1:10000 |
| Anti-mouse IgG | 7076S | Cell Signaling | WB, 1:3000 |
| Anti-rabbit IgG | 7074S | Cell Signaling | WB, 1:3000 |
| CoraLite488-conjugated Goat Anti-Rabbit IgG (H+L) | SA00013-2 | Proteintech | IF, 1:200 |
| Lobaplatin | 135558-11-1 | MCE | 5-80 μg / mL |

**Supplementary figures**

**
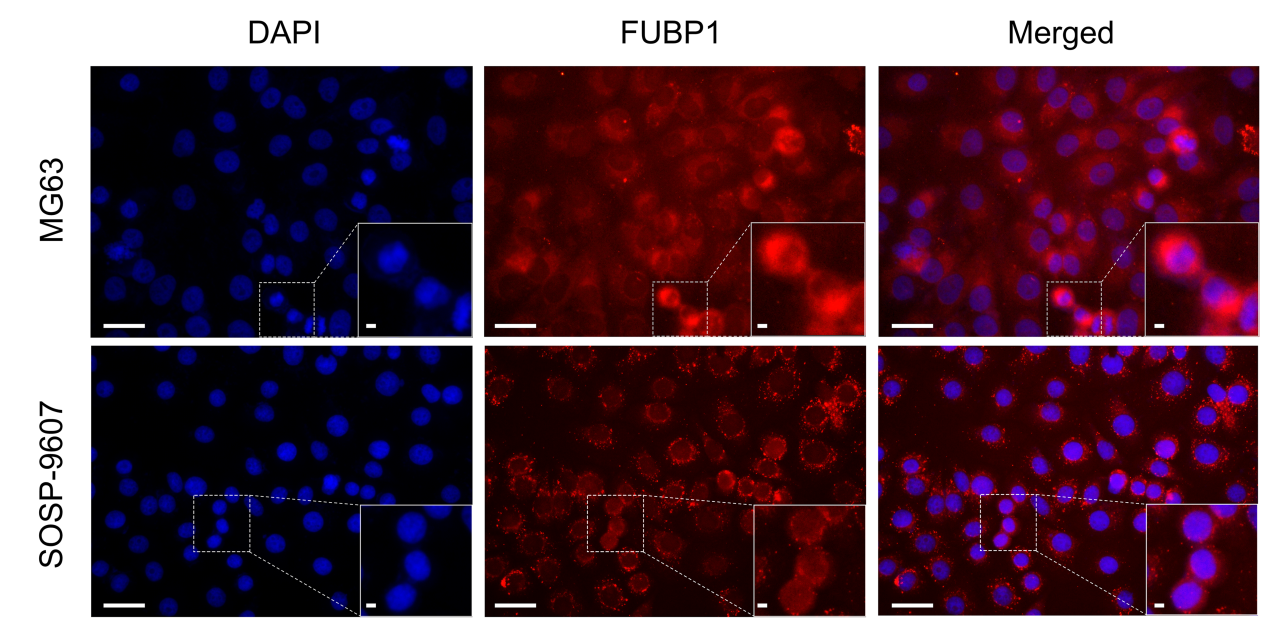
**

**Figure S1.** RNA FISH analysis showed that FUBP1 was predominantly located in the nuclei of MG63 and SOSP-9607 osteosarcoma cells (scale bars=50 μm, scale bars= 3.125 μm).


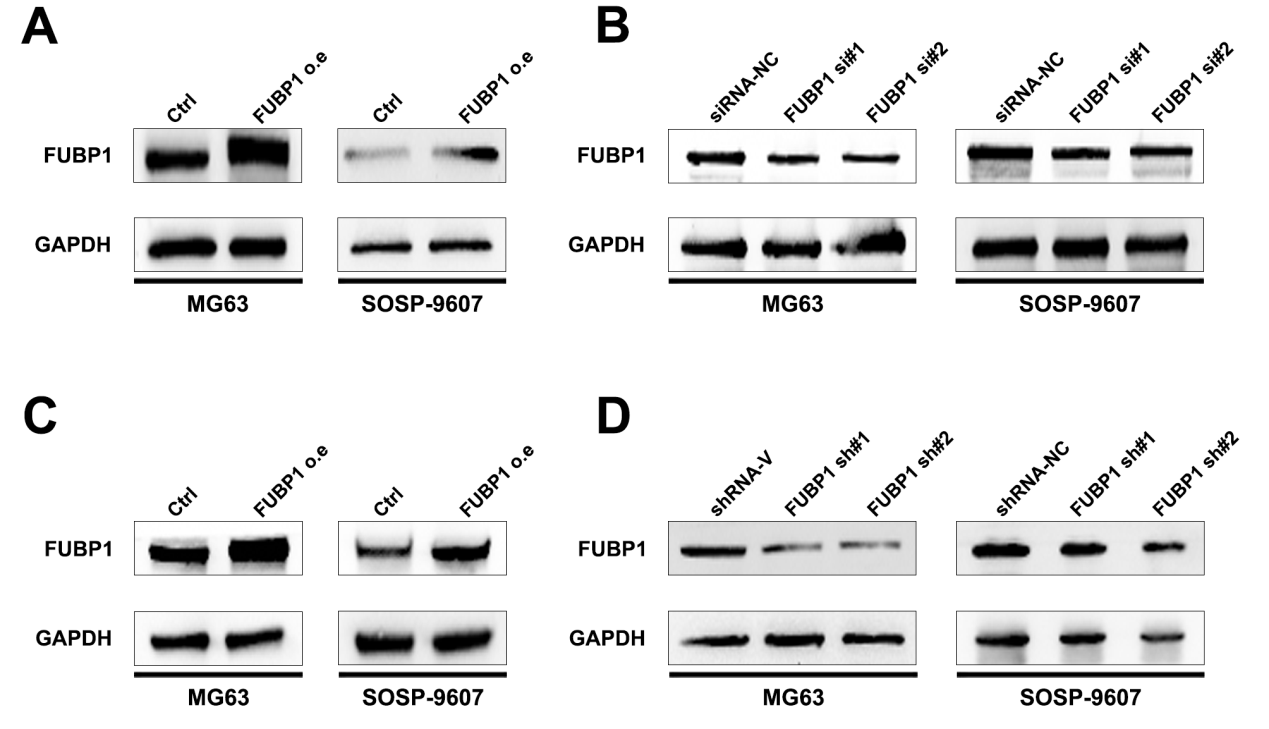


**Figure S2.** Western blotting analysis of FUBP1 levels in the indicated cells. GAPDH was used as a loading control. The expression level of FUBP1 in osteosarcoma cells that were transfected with the overexpression plasmids **(A)** and siRNAs **(B)** for FUBP1. The expression level of FUBP1 in osteosarcoma cells that were infected with lentiviruses stably overexpressing **(C)** and knocking down **(D)** FUBP1.


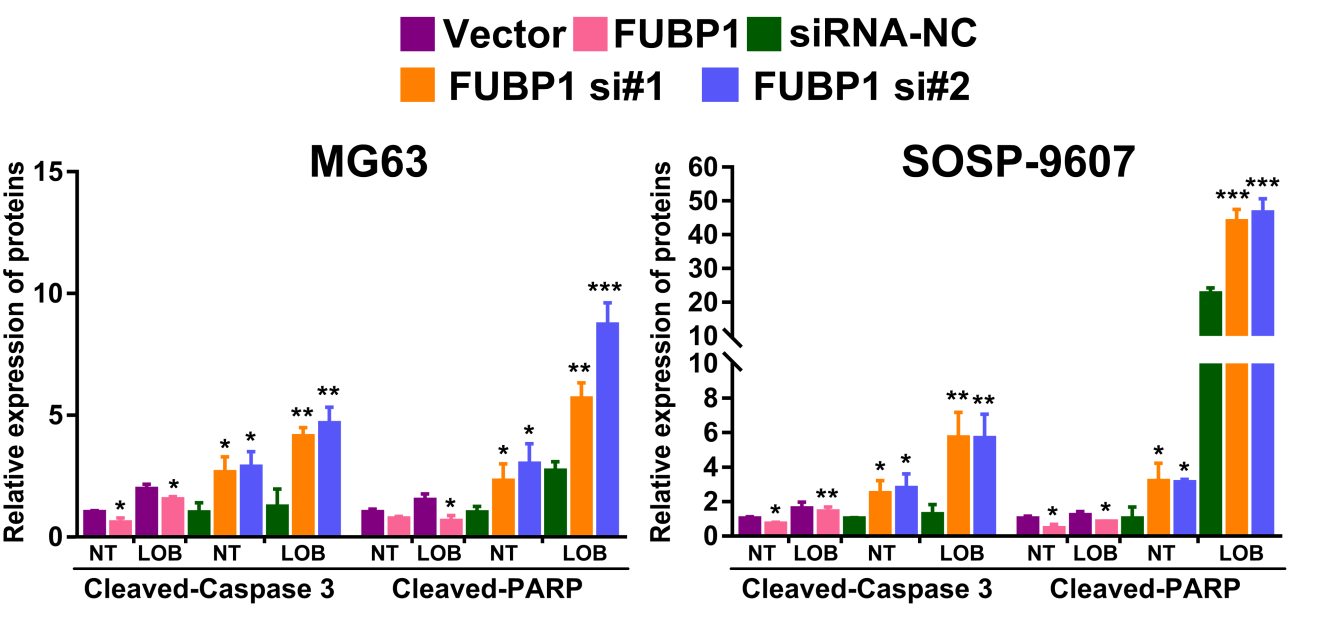


**Figure S3.** Statistical analyses of western blotting results of cleaved caspase 3 and cleaved PARP in the indicated osteosarcoma cells.


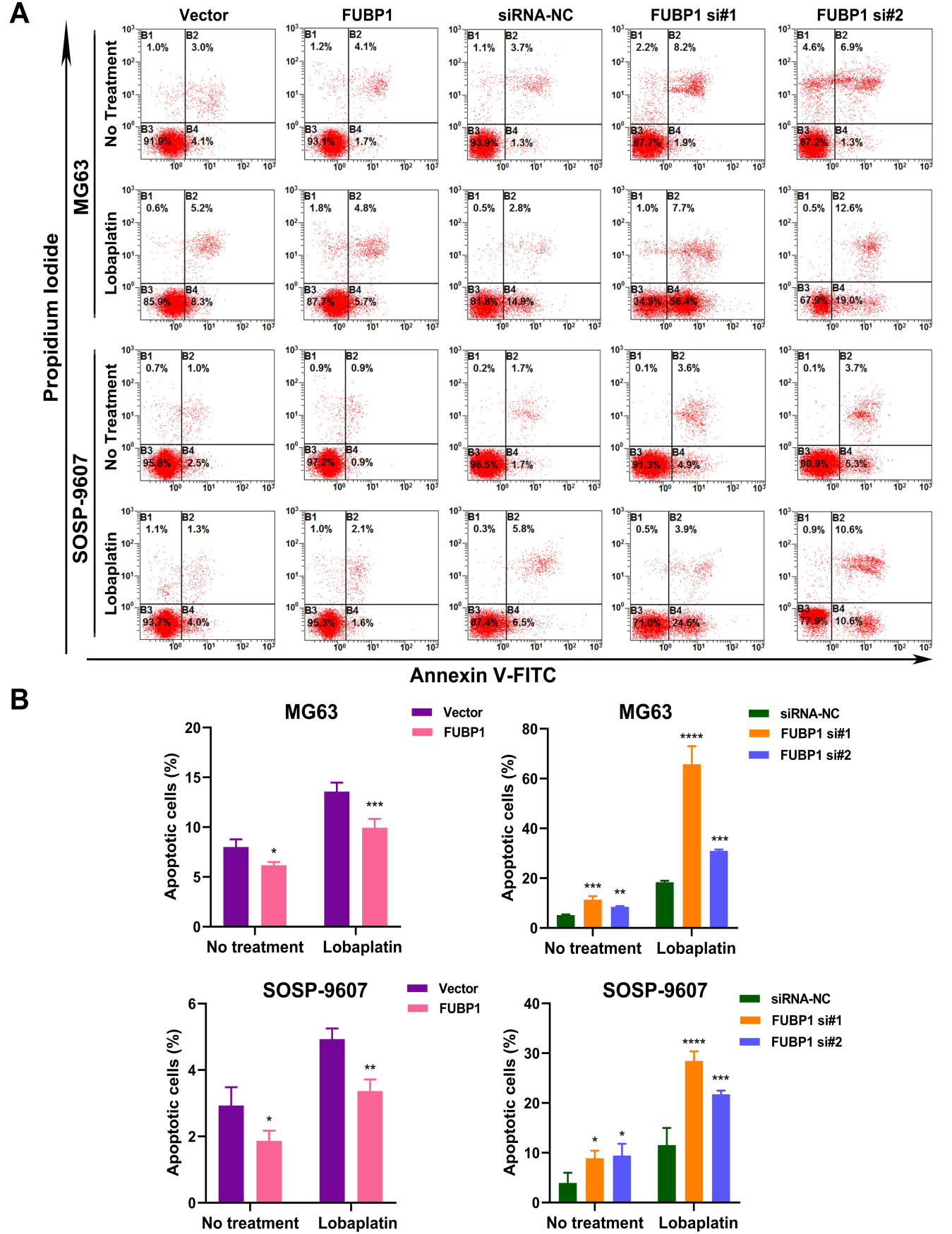


**Figure S4.** The apoptosis rates of MG63 and SOSP-9607 cells treated with or without lobaplatin for 24 h were detected using Annexin V-FITC and PI staining. **A,** Annexin V-FITC and PI staining of the indicated cells. **B,** Statistical analysis of the apoptosis rates in each group. FITC, fluorescein isothiocyanate; PI, propidium iodide.

**
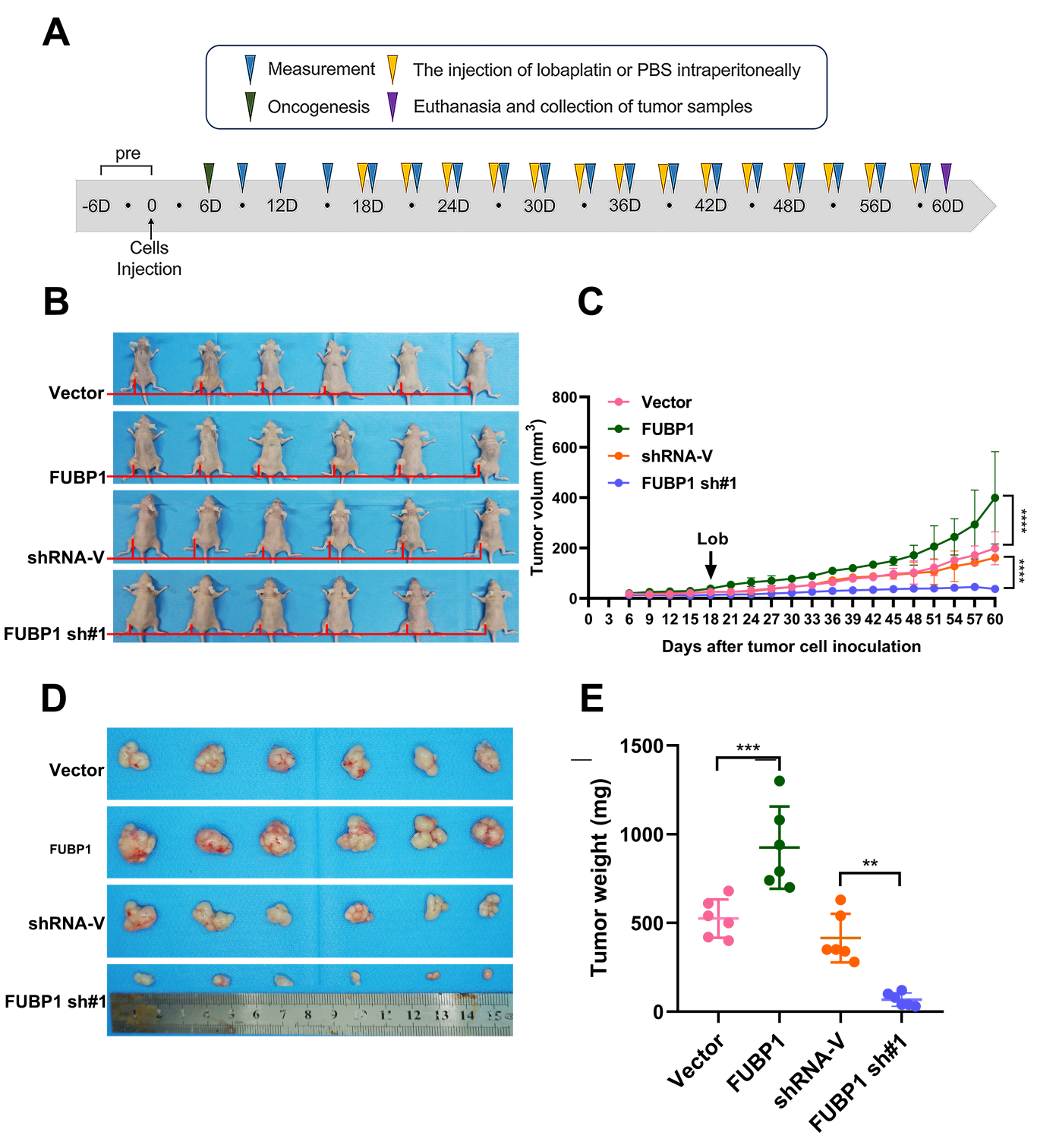
**

**Figure S5.** FUBP1 conferred lobaplatin resistance in SOSP-9607 osteosarcoma cells *in vivo*. **A,** Schematic of the tumor xenograft experiment. The indicated osteosarcoma cells were injected subcutaneously into nude mice, which were then subjected to intraperitoneal treatment with lobaplatin (3 mg/kg) twice per week. **B,** Representative images of tumor-bearing mice inoculated with the indicated cells and then treated with lobaplatin. **C,** The entire tumors were acquired from the nude mice in each group. **D,** Tumor volumes were measured and recorded on the indicated days. **E,** Tumor weights were recorded and are indicated as the means±SEMs.


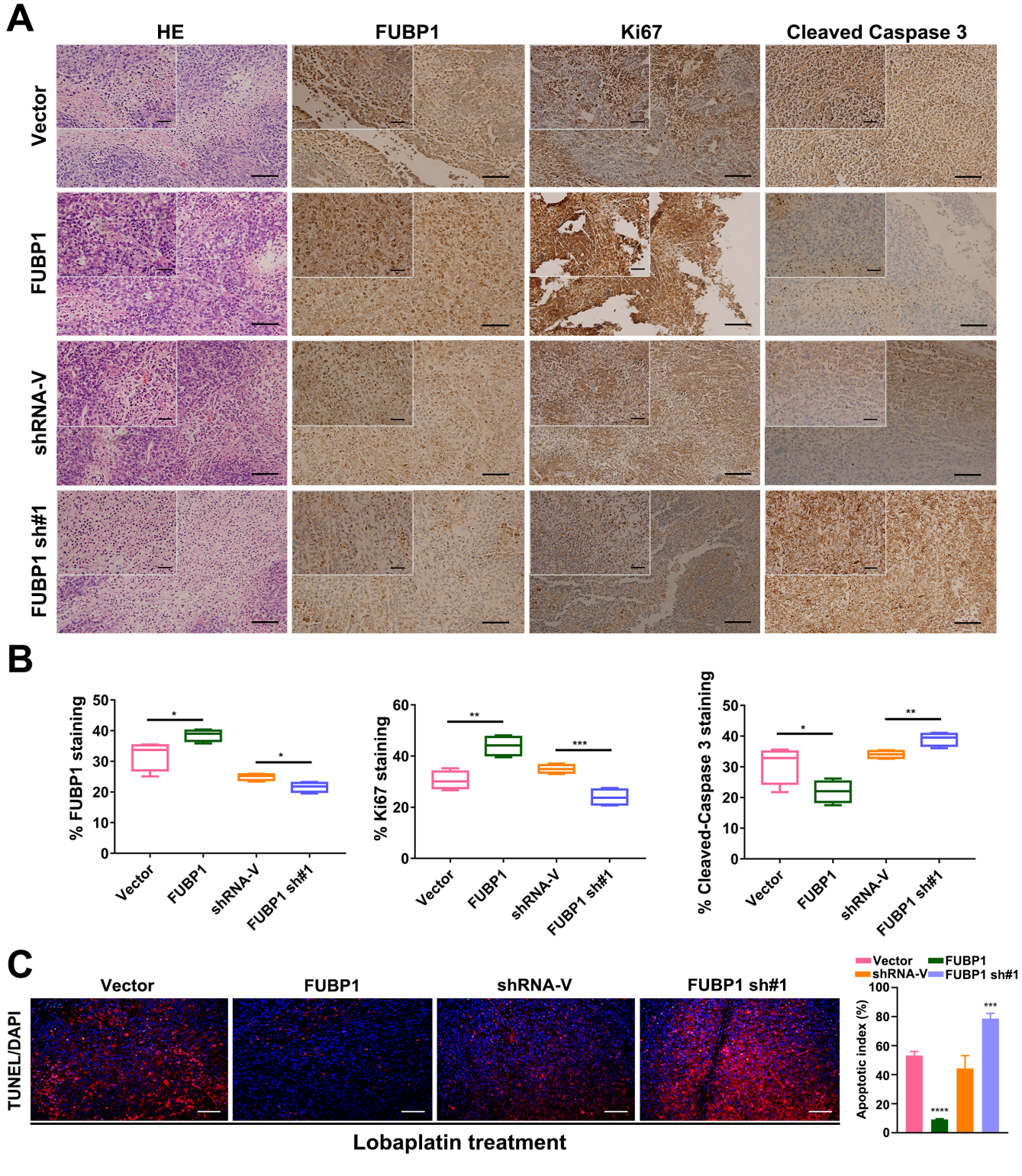


**Figure S6.** Immunohistochemical staining and TUNEL staining of xenograft tumor tissues with SOSP-9607 osteosarcoma cells. **A,** Hematoxylin-eosin staining and immunohistochemical staining with antibodies against FUBP1, Ki67, and cleaved caspase-3 were performed on xenograft tumor tissue sections (×400, scale bars=50 μm; ×200, scale bars=100 μm). **B,** Box-and-whisker plots showing the mean values ± SEMs of Ki67 and cleaved caspase 3 staining from each group of mice (n = 6). **C,** After fixation with paraformaldehyde, TUNEL staining assays demonstrated cell apoptosis in the indicated tissues (scale bars=100 μm). * P <0.05, ** P <0.01, *** P <0.001, **** P <0.0001.


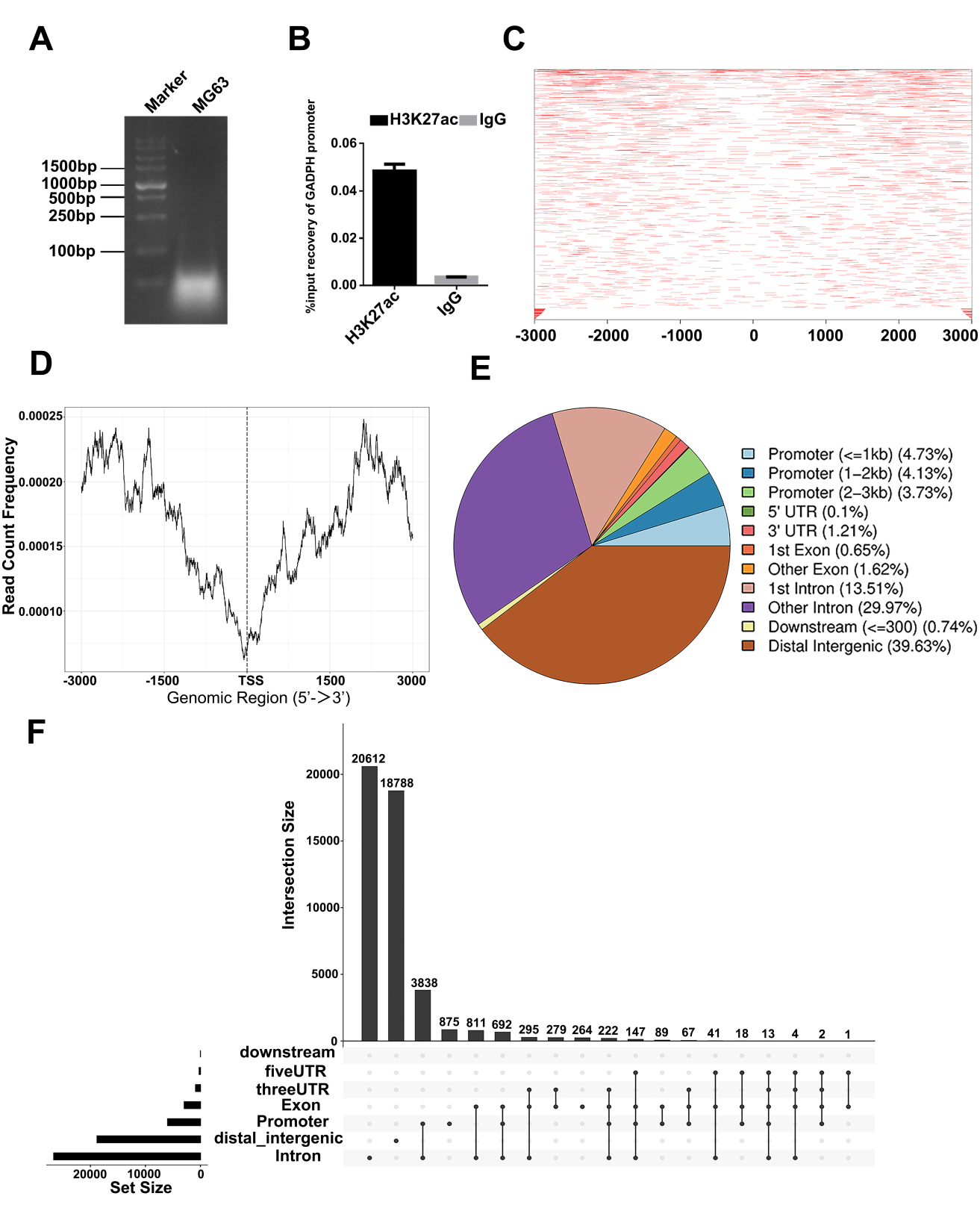


**Figure S7.** The enrichment intensity distribution of reads based on the well-constructed chromatin immunoprecipitation assay system to explore FUBP1-binding DNAs. **A,** Agarose gel electrophoresis showed that most of the DNA fragments caused by chromosome breaks in osteosarcoma cells were concentrated at 100-500 bp. **B,** Real-time PCR analysis of GAPDH promoter levels in the positive control H3K27ac and negative control rabbit IgG. **C,** A heatmap showing the enrichment intensity distribution of reads near the transcriptional start site. **D,** Summary plot for the enrichment intensity distribution of reads near the transcriptional start site. **E,** Peak distribution ratio of gene structural elements. **F,** UpSetplot of peaks on structural elements in genes.


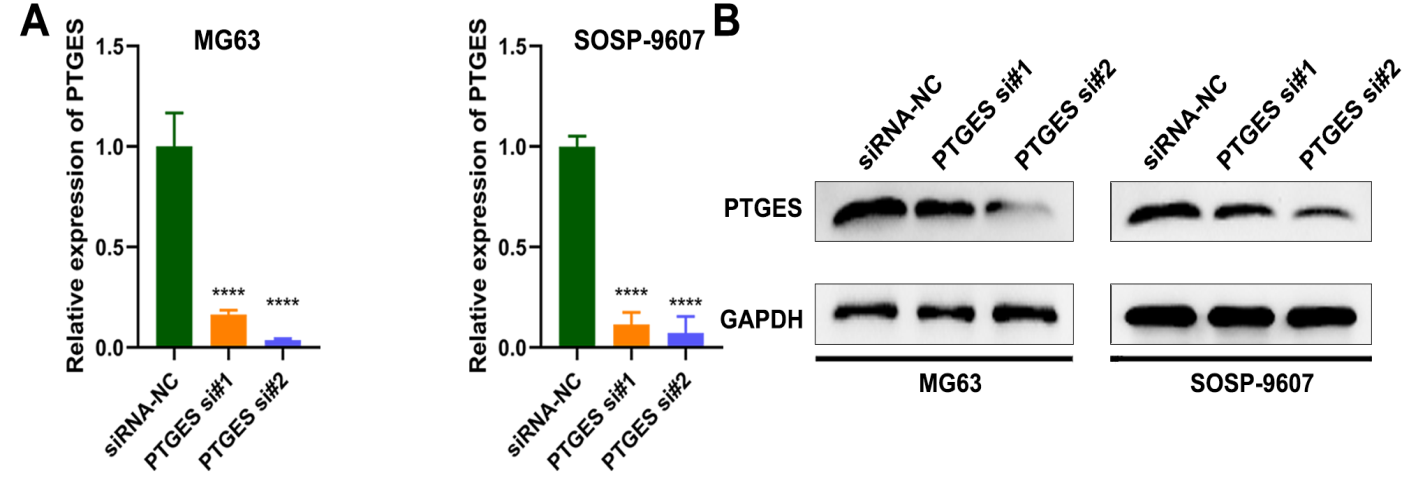


**Figure S8.** Real-time PCR **(A)** and western blotting **(B)** analyses of PTGES levels in the indicated osteosarcoma cells. GAPDH was used as a control.
